# Metabolomic analysis of Yunnan cigar tobacco leaves: impact of geography and climate on flavor characteristics and machine learning-based origin traceability

**DOI:** 10.3389/fpls.2025.1703429

**Published:** 2026-02-18

**Authors:** Yuping Wu, Guijuan Zhao, Yi Li, Guifeng Li, Wenyuan Wang, Lei Yang, Zhonglong Lin, Heng Yao, Fangchan Jiao, Gaokun Zhao, Yongping Li, Guanghai Zhang, Meiwei Zhao, Tao Zhang, Jin Wang

**Affiliations:** 1 Yunnan Academy of Tobacco Agricultural Sciences, Kunming, China; 2 Key Laboratory of Natural Products Synthetic Biology of Ethnic Medicina Endophytes, Yunnan Minzu University, Kunming, China; 3 Yunnan Key Laboratory of Tobacco Chemistry, China Tobacco Yunnan Industrial Co., Ltd., Kunming, China; 4 College of Agronomy, Yunnan Urban Agricultural Engineering and Technological Research Center, Kunming University, Kunming, China

**Keywords:** biomarkers, flavor profile, geographical origin, machine learning, metabolomics

## Abstract

To investigate how Yunnan's distinctive geographical and climatic conditions shape the unique metabolic profile of its cigar tobacco leaves (CTLs), and to establish a reliable method for origin traceability using machine learning, a non-targeted metabolomics analysis was conducted on 71 CTL samples collected from the Dominican Republic, Indonesia, and Yunnan, including Lincang, Pu’er, and Yuxi within Yunnan. A total of 778 highly reliable metabolites were identified. Influenced by Yunnan's high altitude, large diurnal temperature variation, intense ultraviolet radiation, and relative dryness, its CTLs exhibited characteristic metabolic profiles, with significant enrichment in pathways such as flavone and flavonol biosynthesis and betalain biosynthesis. Elevated levels of polyphenols, indoles, jasmonates, carotenoids, and other compounds were linked to Yunnan CTLs' distinct woody, roasted, and astringent flavor profile. Twelve key biomarkers were selected using Multivariate methods with unbiased variable selection in R (MUVR). Machine learning algorithms—including LDA, LR, GMM, KNN, and SVM—were applied to these biomarkers, achieving highly accurate origin discrimination across national (Yunnan vs. Dominican Republic/Indonesia) and regional (Lincang, Pu’er, Yuxi) scales. Validation results showed a median false classification rate of 0.1 over 100 iterations and an AUC close to 1, confirming the model's high accuracy and robustness for CTLs origin traceability.

## 1 Introduction

Cigar, a product of aged and fermented tobacco leaves, has been cherished by smokers worldwide for its unique flavors and aromas (Yao et al., 2024; Wang et al., 2023) Its qualities are attributed to the complex interplay of various chemical compounds formed during the tobacco plant’s growth, harvest, and subsequent processing stages (Li et al., 2021b; Zheng et al., 2022). The unique terroir of different tobacco-growing regions, including soil composition, climate, and agricultural practices, significantly influences the final biochemical profile of the tobacco leaves, thereby contributing to the distinct sensory attributes of cigars (Hu et al., 2023; Yan et al., 2022). Due to the promising geographical and climatic conditions and fermentation process, the current high-quality cigar tobacco is mainly produced in Cuba, the Dominican Republic, Indonesia, the United States, Honduras, etc (Chen et al., 2024). Dominican CTLs are known for their distinctive mellow, smoky, grassy, and smoothness (Chen et al., 2024; Zheng et al., 2022; Geng et al., 2023). Indonesian CTLs were characterized considerably by fruity, and woody, leathery, peppery, and baked aroma (Zheng et al., 2022; Yang et al., 2024). As a high-quality tobacco-producing area in China, the cultivation of cigar tobacco in Yunnan is gradually increasing. Yunnan, mainly in Yuxi, Lincang, Pu’er, have achieved a cigar tobacco cultivation of 1,000 hectares, accounting for more than 50% of China’s.

As the global cigar market expands, there is an increasing demand for more precise and scientific methods to distinguish cigars from different regions based on their chemical composition, offering insight into their origin, quality, and authenticity (Ye et al., 2023). On the other hand, origin authenticity is an important part of product authenticity (Ye et al., 2023). Origin traceability is of great significance for protecting agricultural products’ origin, supervising agricultural products’ circulation, and guaranteeing product quality (Jin et al., 2021). There are many technologies used for origin traceability, such as isotope analysis (Zhi et al., 2023), element analysis (Wang et al., 2021), infrared spectroscopy (Liang et al., 2022), nuclear magnetic resonance (Maestrello et al., 2022), and DNA analysis (Lanubile et al., 2024).

Metabolomics is the comprehensive analysis of small molecules, or metabolites, within a biological sample (Muthubharathi et al., 2021). This technique is based on the premise that the metabolic profile of an organism reflects its genetic makeup, environmental conditions, and physiological state (Pillon et al., 2021). By analyzing the metabolic profile of a sample, it is possible to gain insights into its biological origin and traceability. This approach has emerged as a powerful tool (Li et al., 2021a), allowing researchers to characterize complex biological systems by identifying the metabolites that contribute to specific traits (Muthubharathi et al., 2021; Pillon et al., 2021).

This paper aims to analyze the differences in chemical composition and metabolism of cigar tobacco leaves(CTLs) from Yunnan, Dominica, and Indonesia using metabonomics and reveal the material basis of the flavor profile of CTLs from different geo-origins. To further understand the shaping and influence of geographical climate on the flavor characteristics of CTLs in Yunnan. In addition, metabolites combined with machine learning methods were used to accurately trace the origin of CTLs at different geographical scales, from national to regional levels.

## 2 Materials and methods

### 2.1 Sampling

71 cigar leaf samples were collected from dominica (DMNJ, n=16), Indonesia (IDA, n=13), Yunnan, China (CN, n=42). China’s Yunnan cigar tobacco mainly comes from the three major cigar tobacco producing areas in Yunnan, Lincang(LC, n=10), Pu’er (PE, n=12)and Yuxi (YX, n=20). Dominican samples were collected in Cibao Valley regionand Indonesian samples were collected in Sumatra Island. The Yunnan samples were representative of the core cigar-producing regions: Lincang, Pu’er, and Yuxi. Cigars from these three places have the unique style of Yunnan cigars. The varieties of Indonesian samples are BESUKI and SUMATRAL, while the Dominican cigar varieties include HABANO, OLOR, and CONNECTICUT. Varieties of Yunnan samples are YX1, YX2, and YX8, which have been introduced from abroad and improved. All samples were collected during the 2020–2022 production season to minimize seasonal variation.

The following is the processing technology of Yunnan cigars. Referring to the processing technology of Cuban cigars, fresh tobacco leaves, after being harvested, enter the air-curing process, and the specific process is as follows: 1. Withering period. Duration days: 2–4 days, temperature is 25°C-28°C, relative humidity is about 80%. 2. Yellowing period. Duration days: 5–10 days, temperature is 25°C-28°C, relative humidity is about 85%. 3. Browning period. Duration days: 10–15 days, temperature is 28°C ~ 30°C, relative humidity is 85%-80%. 4. Dry tendon period. Duration days: 7–10 days for tobacco planted in shade (7–15 days for tobacco planted in sunshine). Tobacco leaves planted in shade should have a temperature of 30°C-32°C and a relative humidity of 80%-35%. Tobacco leaves planted in the sun require a temperature of 32°C ~ 35°C and a relative humidity of 80% -35%. After that, it enters the fermentation process, and the treatment method refers to “DB53/T1303–2024 Technical Regulations for Agricultural Pile Fermentation of Cigar Tobacco Leaves”. Finally, the storage conditions are the relative humidity 70-75% and temperature 20 ± 2°C.

Prior to analysis, all cigar leaf samples were dried in a constant temperature oven at 40°C for 48 hours until a constant weight was achieved. The dried samples were then ground into powder and passed through a 40-mesh sieve to ensure homogeneity.

### 2.2 Non-targeted metabolic analysis

Non-targeted metabonomic analysis and subsequent data analysis were undertaken by BGI (Wuhan, 430070, China). The sample extraction and determination methods were similar to those of Li et al. (Li et al., 2024), and some methods were adjusted. After the sample was extracted, d3-Leucine(250ng/ml), 13C9-Phenylalanine(25μm/ml), d5-Tryptophan(12.5μm/ml), 13C3-Progesterone (12.5μm/ml)were added as internal standards, UPLC-MS/MS detection was performed. The off-line data of mass spectrometry were imported into the Compound Discoverer 3.3 (Thermo Fisher Scientific, USA) software, combined with BMDB (BGI metabolome database), mzcCloud database and ChemSpider online database for mass spectrometry data analysis.

### 2.3 Data analysis

The PCA and PLS-DA were performed using SIMCA 14 (sartorius Stedim, Germany). Analysis of metabolic differential compounds was performed using the Metware Cloud, a free online platform for data analysis (https://cloud.metware.cn). Metaboanalyst 6.0 was used for metabolic pathway enrichment analysis(https://www.metaboanalyst.ca). The volcano plot, cluster heatmap, box plot, RDA etc. are mapped using Metware Cloud. For metabolic indices discovery, we used multivariate methods with Unbiased Variable selection in R package (MUVR) to select potential metabolic indices (Shen et al., 2021; Wang et al., 2023). The algorithm and multi classification ROC curve are also implemented by R package.

## 3 Results and discussion

### 3.1 Overview of origins geographical and climatic characteristics

Plants are highly sensitive to the environment in which they grow. Geographic and climate conditions, such as temperature, precipitation, altitude, and soil, have profound impacts on plant growth, development, and metabolic processes (Chaudhary et al., 2022). **Table 1** presents the geographical and climatic data of three countries’ main cigar-producing areas. As shown in **Table 1**, the rainfall in Yunnan, China, has relatively low, the humidity and the temperature is relatively low, the altitude is high, and the temperature difference between day and night is significant; Indonesia has high rainfall and humidity, slight temperature difference between day and night, and the soil is volcanic ash soil; Dominica is red soil with moderate rainfall and other factors similar to Indonesia. Geographic and climate conditions play a critical role in shaping plant metabolism, influencing both primary and secondary metabolite production. Increased ultraviolet radiation associated with high altitude is correlated with enhanced metabolism and accumulation of flavonoids in plants, as reported in previous studies (Pant et al., 2021) A significant temperature difference is beneficial for the accumulation of carbohydrates, which has been confirmed in our previous work (Wu et al., 2023). The research shown that Yunnan’s CTLs has the highest total sugar, reducing sugar, and starch content than Indonesian and Dominican CTLs (Wu et al., 2023). An appropriate temperature is beneficial for plant growth and photosynthesis. And relatively high or low temperatures have a stress effect on plants (Yamori et al., 2022). Red soil is rich in Fe (Zong et al., 2021), volcanic soil is rich in Ca, Mg, Zn and other trace elements (Mulyono et al., 2024; Abe et al., 2020), both of which have important effects on plant metabolism. In conclusion, geographic and climate conditions play a critical role in shaping cigar tobacco metabolism. Moreover, agricultural production practices, fertilization, varieties, and fermentation processes also have significant impacts on the metabolites of cigar tobacco leaves.

**Table 1:** The geographic and climate characteristics of cigar tobacco origins (Mulyono et al., 2024; Liang et al., 2021; Delanoy et al., 2022; Kettler, 2022, Jin et al., 2012, Irfan et al., 2023).

Origins	Soil	Climate	Rainfall** (mm)	Temperature** (°C)	Day-night temperature difference** (°C)	Humidity** (%)	Elevation (m)
DMNJ(Cibao valley)*	red soil	tropical marine climate	1200-2000	25-30	3-7	>80	50-900
INA(Sumatra)*	volcanic (ash) soil	tropical rainforest climate	1600-2200	25-30	3-5	>80	10-700
CN(Yunnan)*	red soil	subtropical mountain monsoon	400-1500	10-28	10-15	40%-80	1200-2200

*The data is of mainly cigar-producing areas in brackets.

**It’s growing season data.

### 3.2 Multivariate statistical analysis

**Figure 1:**
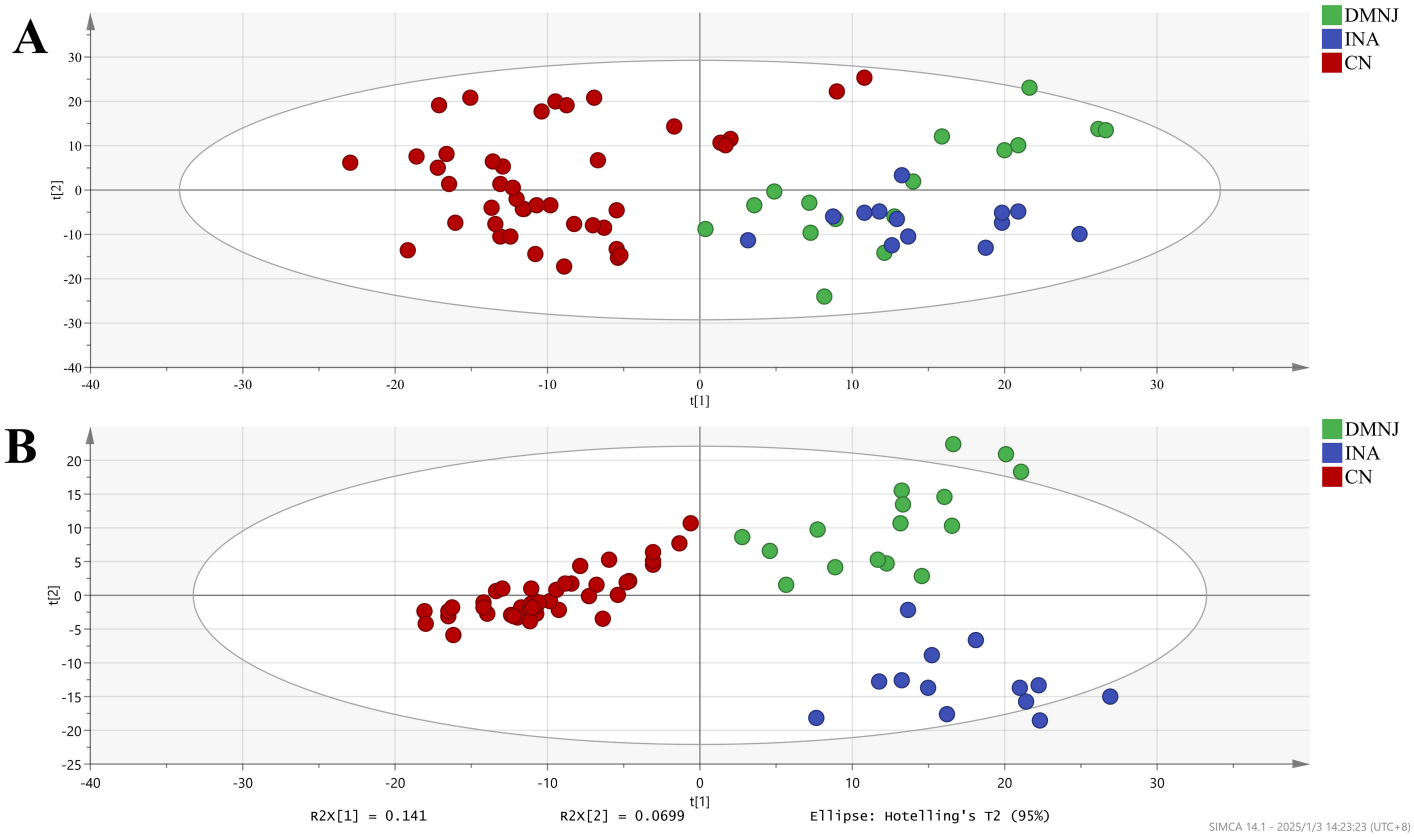
The PCA **(A)** and PLS-DA **(B)** plots of CTLs form Dominica, Indonesia and China (Yunnan).

High-resolution LC-MS/MS identified 778 highly reliable metabolites, with 494 compounds identified in the positive mode and 284 compounds identified in the negative mode. A total of 433 metabolites (55.5%) were successfully annotated by matching against the PubChem and Kyoto Encyclopedia of Genes and Genomes (KEGG) database. Principal Component Analysis (PCA) is a commonly used unsupervised data dimensionality reduction method. From the PCA shown in **Figure 1A**, it can be seen that there are significant differences between Yunnan and Indonesian and Dominican CTLs, but Indonesian and Dominican CTLs have a certain degree of overlap, indicating that there may be some similarity between their metabolite fingerprints, making it difficult to distinguish.

And Partial Least Squares Discrimination Analysis (PLS-DA) is a commonly used supervised data dimensionality reduction method. Now, PLS-DA is applied to distinguish CTLs from different origins. As shown in **Figure 1B**, CTLs from the three regions can be clearly distinguished. The (R2 Y) and (Q2) of the PLS Da model are 0.812 and 0.745, respectively (see **Supplementary Figure S1A**), indicating good performance in identifying the origin of cigar tobacco leaves. R2 Y and Q2 parameters greater than 0.5 and close to 1 indicate that the model has a high correlation (R2 Y) and predictive ability (Q2) for sample geographic differentiation (Rivera-Pérez et al., 2021).

Furthermore, PLS-DA analysis showed that the number of important variables (VIP values) greater than 1 in the projection reached 493 (as shown in **Supplementary Figure S1B**). A VIP value >1 indicates that the variable is important to the PLS-DA model. If the independent variable and Y have the same explanatory power, the VIP value will be 1. The higher the VIP value, the greater the contribution of the independent variable to the model. Therefore, the relatively high proportion of VIP>1 (about 39.5%) also reflects the significant similarity in metabolites of cigar tobacco leaves from these three production areas, which to some extent reflects the difficulty in distinguishing CTLs production regions (Zhao et al., 2022).

### 3.3 Metabolomics analysis

The CTL Metabolomics analysis was performed using the MetaboAnalyst 6.0 (https://www.metaboanalyst.ca/home.xhtml) and the Metware Cloud (https://cloud.metware.cn). The significant differential metabolites are metabolites with P <0.05, fold changes of >2 or <0.5, and VIP>1, and visualized by Volcano plots. The significant differential metabolites were used for clustering analysis in a heatmap, followed by a pathway enrichment analysis with metabolites using Arabidopsis thaliana (thale cress) (KEGG) pathway library. The significantly enriched pathway threshold is -lg(P)>1 and pathway impact>0.25.

#### 3.3.1 Comparison of the metabolic profiles of Dominican and Yunnan CTLs

**Figure 2:**
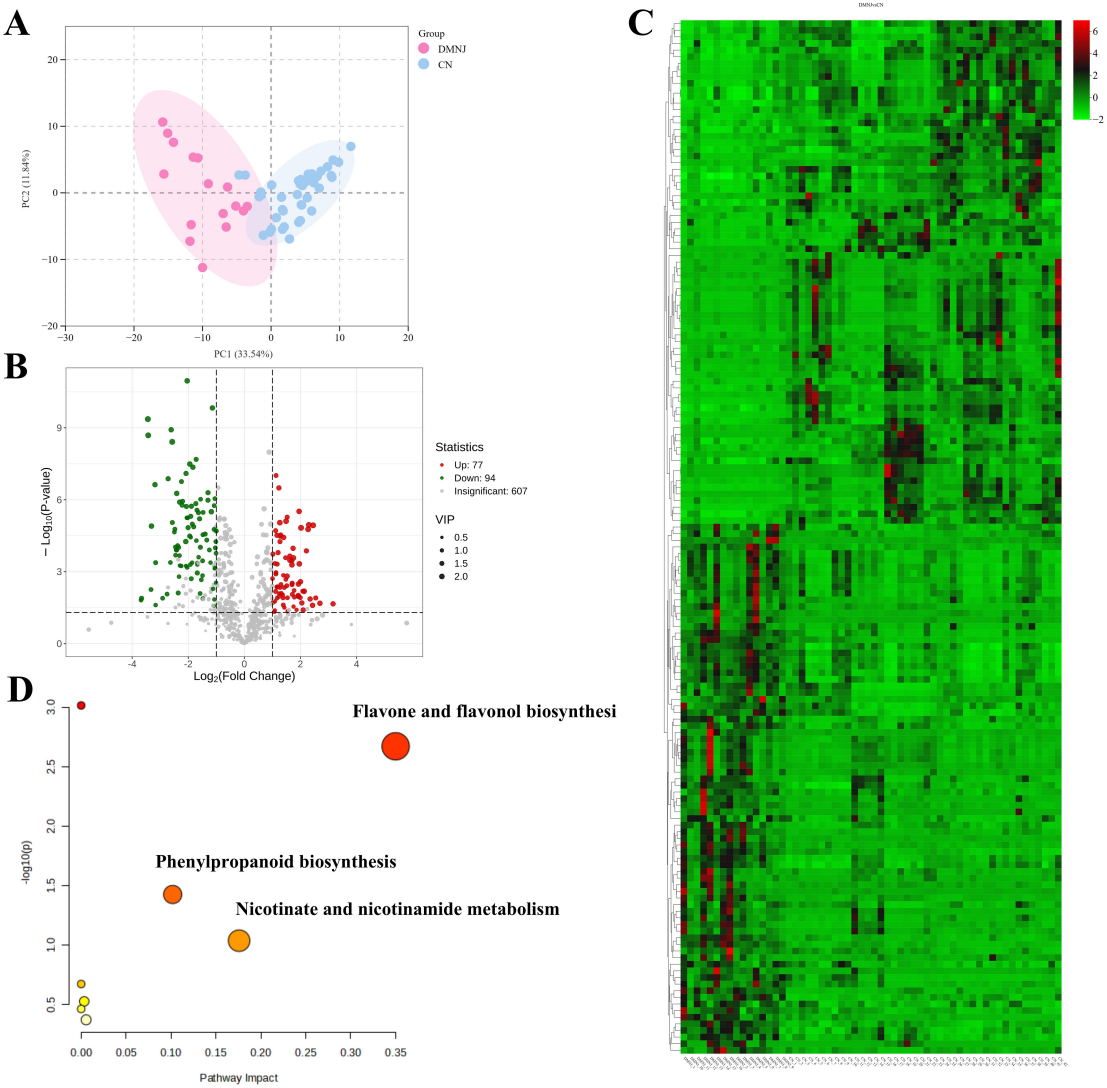
Metabolic profiles of Dominican (DMNJ) and Yunnan (CN) CTLs. **(A)** Plot of PCA. **(B)** Volcano plot of differential metabolites. **(C)** Heatmap of differential metabolites. Red indicates metabolites that were up-regulated and green indicates metabolites that were down-regulated. **(D)** Pathway enrichment plot. Colors represent the relative degree of the impact of each pathway (X-axis) and statistical significance (Y-axis).

PCA can distinguish between Dominican and Yunnan CTLs with little overlap, as shown in **Figure 2A**. Through differential metabolite analysis, 171 significantly different metabolites were identified, with 77 metabolites being significantly up-regulated and 94 metabolites being significantly down-regulated, as shown in the volcano plot in **Figure 2B** and **Supplementary Table S1**. Cluster analysis was conducted on 203 differential metabolites, which can significantly distinguish Dominican and Yunnan CTLs, as shown in **Figure 2C**.

**Figure 3:**
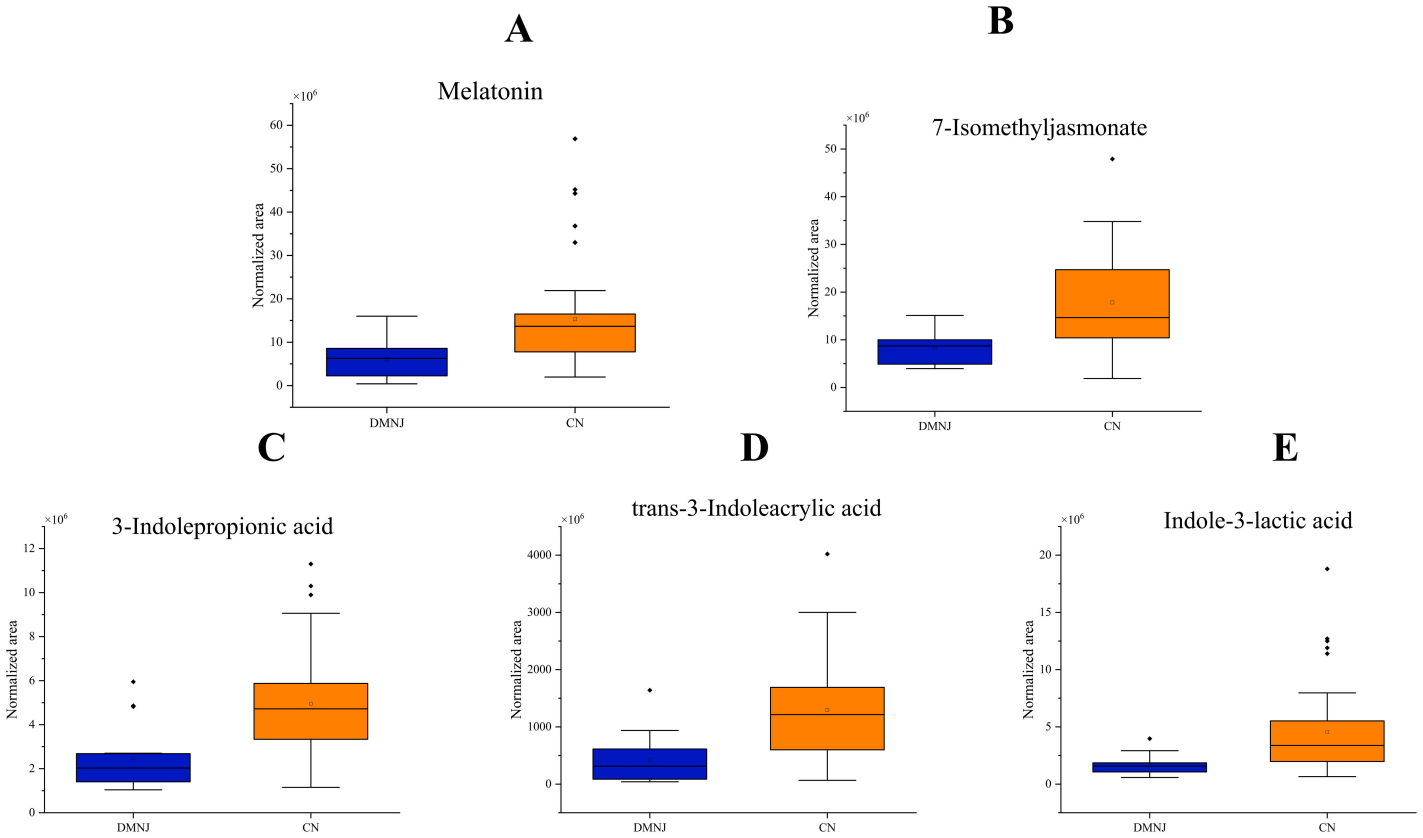
Box plots labeled **A–E** compare the normalized area of various compounds between DMNJ and CN groups. **A** shows melatonin, **B** shows 7-Isomethyljasmonate, **C** shows 3-Indolepropionic acid, **D** shows trans-3-Indoleacrylic acid, and **E** shows Indole-3-lactic acid. CN group exhibits higher values than DMNJ across all compounds. Each plot includes outliers.

Through differential metabolic analysis(as shown in **Supplementary Table S1**), it was found that the content of indole compounds such as 3-Indolepropionic acid, Indole-3-lactic acid, trans-3-Indoleacrylic acid was relatively high in Yunnan CTLs(as shown in **Figure 3**). Indoles are one of the plant growth factors that can promote plant growth, cell division, and root growth (Sun et al., 2023). They can also be used to resist external stress. Therefore, due to the relatively dry weather in Yunnan, cigars require a relatively high content of indoles to promote root growth. However, some may also be in response to high UV radiation stress in Yunnan. In addition, the content of 7-Isomethyljasmonate is also relatively high(as shown in **Figure 3**). As a plant hormone, the increase in jasmonic acid content may also be related to the relative drought and UV stress in Yunnan (Raza et al., 2021). Furthermore, indoles and jasmonic acid compounds also have a certain odor (Acree et al., 1985; Priolo et al., 2009) and maybe contribute to the flavor of cigars. In Yunnan CTLs, such as Caffeic acid, Sinapinic acid, Gallic acid, Ferulic acid, Matairesinol, Kaempferol, Rutin, and other polyphenols have high content. Polyphenol content is very important for the quality of cigars. Fermentation with polyphenol oxidase (PPO)-producing microbes specifically degrades polyphenols to form quinones and other substances (Meng et al., 2022), which make the cigar less “green/leafy” harshness, more mellow body, darker “cured” aroma and improves cigar aroma richness while reducing miscellaneous/irritating notes (Zhang et al., 2023). Furthermore, during smoking, heat (up to 900°C) transforms precursors. Polyphenols pyrolyze into phenols (e.g., guaiacol via cleavage) (Rigling et al., 2023), detected by olfactory receptors for smoky sensations (Juhlke et al., 2017). The high content of polyphenols in Yunnan CTLs is potentially associated with the intense ultraviolet radiation in the region, consistent with the known role of polyphenols in plant UV stress response (Shen et al., 2022). Some works reported that the presence of polyphenols in wine can increase its astringency and flavor richness (Soares et al., 2017; Gawel, 1998). Therefore, Sinapinic acid with bitterness (Nandasiri et al., 2021), Gallic acid with bitterness, sourness, and astringency (Robichaud and Noble, 1990), as well as the important flavor compound Rutin in tobacco (Chen et al., 2021), may also increase the astringency and flavor of Yunnan tobacco. In addition, the increase in the content of carotenoids such as 1 ‘- Hydroxy-gamma-carotene and 1,1’ - Dihydroxylycopene in Yunnan CTLs may also be related to the high UV intensity in Yunnan (Badmus et al., 2022). Carotenoids are also important flavor compounds (Vogel et al., 2010) and have a certain color (Gordon et al., 1983), therefore, they have an impact on the flavor and color of Yunnan CTLs. The melatonin content of CTLs in Yunnan is 2.5 times that of the Dominican Republic, which is consistent with the potential influence of Yunnan’s intense ultraviolet radiation on the metabolism of cigar tobacco leaves. In Arabidopsis thaliana, melatonin not only acts as an antioxidant to promote UV-B stress resistance, but also regulates expression of several key components of UV-B signalling pathway to protect the plant from UV-B stress (Yao et al., 2021). After UV-B exposure, melatonin concentration increases in Malus hupehensis (Wei et al., 2019), and it can reduce DNA damage in Nicotiana sylvestris plants (Zhang et al., 2012). Therefore, the increase of melatonin may be related to UV radiation in Yunnan.

Our previous research has shown that the aroma of Yunnan cigars is mainly woody, roasted, fresh sweet, bean, and scored (Wu et al., 2023). And the high content of Tolylacetate [sweet odor (Liu et al., 2024)], Benzoin (sweet-balsamic odor of vanilla) (Fernandez et al., 2006) and alpha-Ionone [(fruit, floral (Aloum et al., 2020)] maybe have significant contributions to the flavor of Yunnan CTLs. Dominican CTLs were reported tohad aromatics of mellow, smoky, grassy, and smoothness. And Dominican CTLs has relatively high content of Hydrocinnamic acid [floral (Stavrou et al., 2024)], trans-Cinnamic acid [warm spicy and balsamic odour (Miyazawa et al., 1996)]. These flavor compounds may be the basis of the flavor compounds in Dominican CTLs.

The KEGG pathway database is the most commonly used database in the study of metabolic and regulatory pathways. The analysis of metabolic pathways helps to systematically understand the impact of the habitat environment on plants, providing a good reference for further exploring the adaptability of plants to the environment. 68 (40%) of the differential metabolites were successfully annotated onto the KEGG pathway for comparison by importing them into Metaboanalyst. KEGG metabolic pathway enrichment analysis identified 8 metabolic pathways, with 3 significantly enriched pathways Flavone and Flavonol biosynthesis, Phenylpropanoid biosynthesis and Nicotinate and nicotinamide metabolism as shown in **Figure 2D**. Flavonoids and flavonols are important polyphenols in plant secondary metabolites, belonging to the two major subclasses of flavonoids. Flavor and flavor compounds can absorb ultraviolet light. At the same time, due to the electron-donating effect of phenolic hydroxyl groups, they can eliminate free radicals and reduce oxidative stress. Thus they play a protective role in plant cells under UV radiation stress. They are important regulatory means for plants to cope with UV stress (Shen et al., 2022; Jaakola and Hohtola, 2010). Phenylpropanoid metabolism is one of the core pathways of plant secondary metabolism, which is involved in the production of a series of important plant compounds, including lignin, flavonoids, isoflavones and phenolic acids (Dong and Lin, 2021). Phenylpropanoid metabolism is the upstream metabolic pathway of flavor and flavor metabolism. It is also an important means for plants to cope with UV radiation stress (Rahim et al., 2023). The enrichment of these two pathways is closely correlated with the high intensity of ultraviolet radiation in Yunnan, which may reflect an adaptive metabolic response of CTLs to the local environment.

Nicotinic acid and nicotinamide metabolism are important secondary metabolic pathways in plants. They not only directly regulate the content of alkaloids in tobacco, but also directly participate in the synthesis and circulation of nicotinamide adenine dinucleotide (NAD) and its phosphorylated form (NADP). NAD/NADP is an important redox coenzyme involved in redox reaction, energy metabolism, cell respiration, photosynthesis and carbon fixation (Hashida et al., 2016; Ashihara et al., 2020). The enrichment of nicotinate and nicotinamide metabolism may be the reason for the high nicotine content in Yunnan CTLs, which may be related to geography and climate, and may also be affected by agricultural production, such as the application of nitrogen fertilizer. In addition, the high carbohydrate content of Yunnan CTLs may also be related to nicotinate and nicotinamide metabolism.

#### 3.3.2 Comparison of the metabolic profiles of Indonesian and Yunnan CTLs

**Figure 4:**
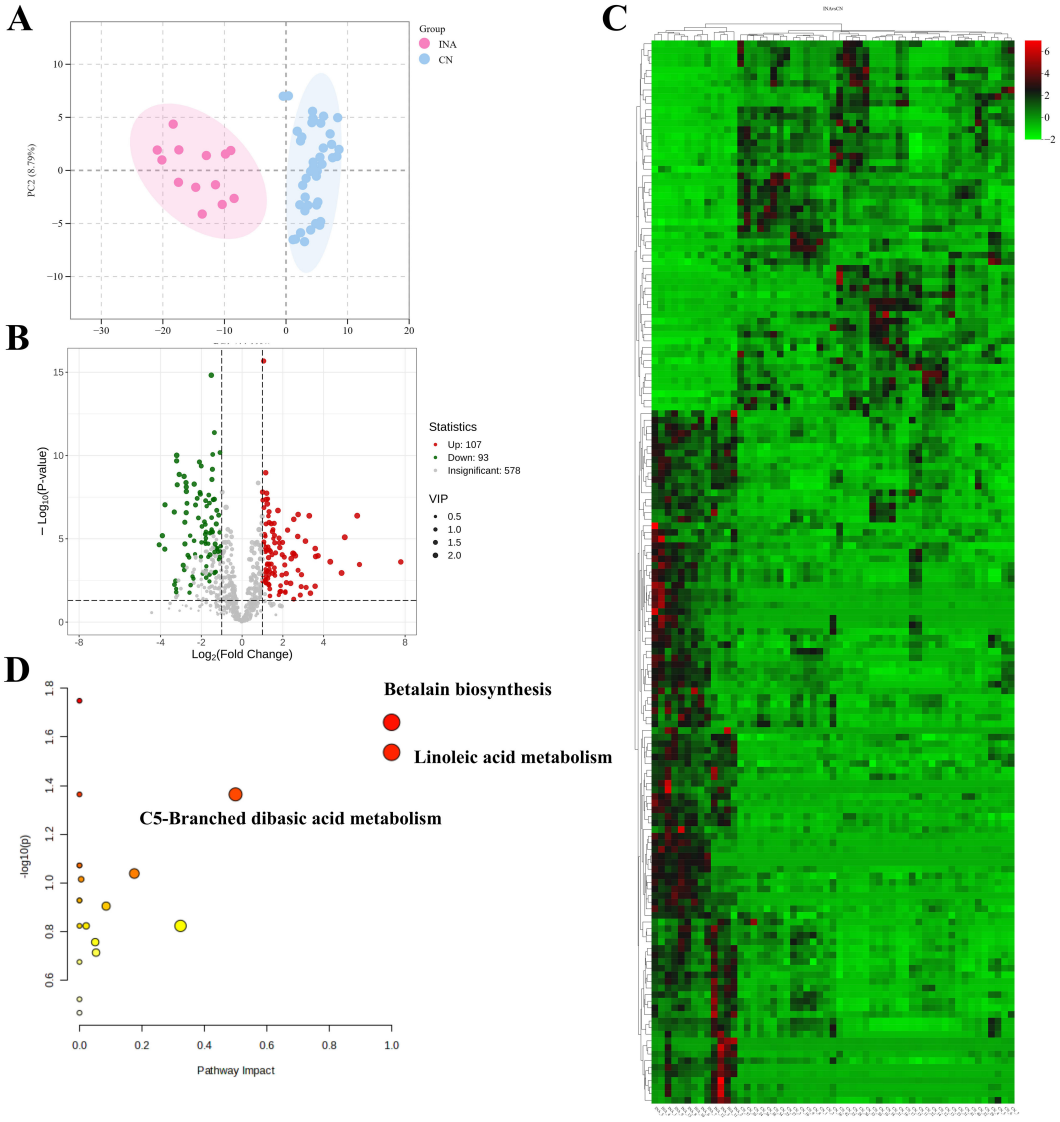
Metabolic profiles of Indonesian (INA) and Yunnan (CN) CTLs. **(A)** Plot of PCA. **(B)** Volcano plot of differential metabolites. **(C)** Heatmap of differential metabolites. Red indicates metabolites that were up-regulated and green indicates metabolites that were down-regulated. **(D)** Pathway enrichment plot. Colors represent the relative degree of the impact of each pathway (X-axis) and statistical significance (Y-axis).

PCA can clearly distinguish Indonesian and Yunnan cigars(as shown in **Figure 4A**). Through differential metabolite analysis, 200 significantly different metabolites were identified, with 107 metabolites being significantly up regulated and 93 metabolites were significantly down regulated. As shown in the volcano plot in **Figure 4B** and **Supplementary Table S2**. Cluster analysis was conducted on 161 differential metabolites, which can significantly distinguish CTLs from these two production areas, as shown in **Figure 4C**.

**Figure 5:**
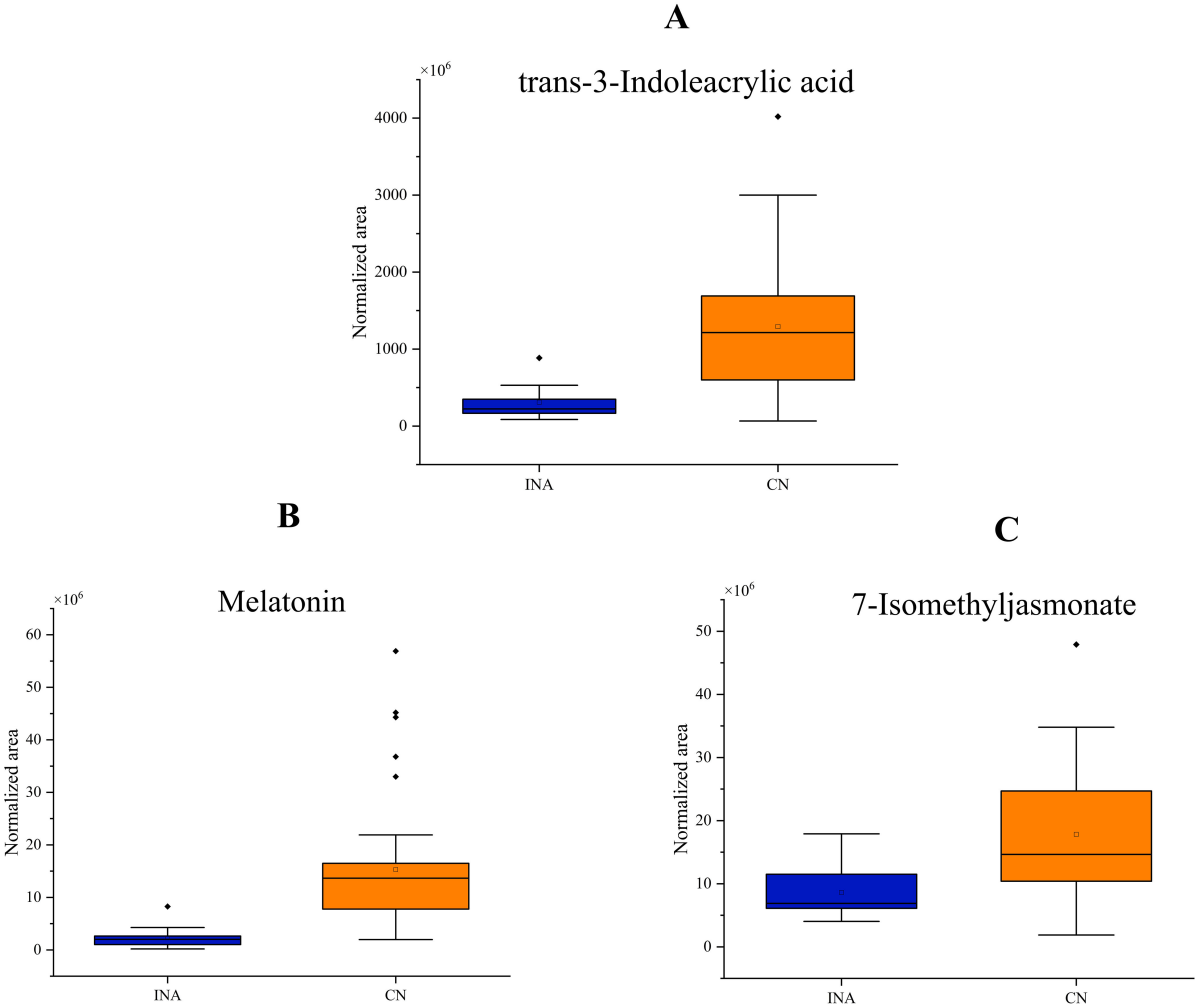
Box plots comparing the normalized area of three compounds between INA and CN groups. **(A)** Trans-3-Indoleacrylic acid shows higher values in CN. **(B)** Melatonin displays greater variability and higher values in CN. **(C)** 7-Isomethyljasmonate also shows higher values in CN than INA. Outliers are marked with dots.

Differential metabolite analysis showed that the levels of 7-Isomethyljasmonate, trans-3-Indoglycolic acid, and melatonin in CTLs from Yunnan were still higher than those in Indonesia (as shown in **Figure 5**). This result aligns with the potential association between high ultraviolet radiations in Yunnan on the metabolism of cigar tobacco. Indonesian CTLs were reported to characterize by leather, pepper, and baked aroma. High content of Hydrocannamic acid, trans-Cannamic acid, Methylcinnamate [fruity, sweet-balsamic (Padalia et al., 2017)], Phenyllacetic acid [honey (Szudera-Kończal et al., 2023)] in Indonesian CTLs, which may be the partial material basis of its flavor.

Comparison of differential metabolites revealed that 65 (33%) were successfully annotated onto the KEGG pathway. KEGG metabolic pathway enrichment analysis identified 20 metabolic pathways, with 3 significantly enriched pathways including Betalain biosynthesis, Linoleic acid metabolism, C5-Branched dibasic acid metabolism, etc., as shown in **Figure 4D**.

Betalains are hydrosoluble pigments almost wholly restricted to species of the core Caryophyllales and, within the fungal lineage, the Basidiomycetes (Tossi et al., 2021). A large number of Basidiomycetes are detected in Yunnan CTLs, which are relatively higher than Indonesian. The difference in Basidiomycetes content may be one reason for the enrichment of the Betalain biosynthesis pathway (Zheng et al., 2022).

Linoleic acid is a kind of polyunsaturated fatty acid, which plays various important roles in plants. It is not only an important part of membrane lipids, but also produces a series of bioactive signal molecules and secondary metabolites through its metabolic pathway, which plays a key role in plant growth and development and environmental stress response. Linoleic acid can metabolized into jasmonic acid (JA), which plays a key role in the response of plants to biotic and abiotic stresses (Nie et al., 2024). Oxidized lipids produced during linoleic acid metabolism can be used as antioxidant signal molecules. Enhance the antioxidant capacity of plants and protect plant cells from oxidative damage (Mata-Pérez et al., 2017). Linoleic acid metabolism involves plant secondary metabolic pathways, such as flavonoid and alkaloid biosynthesis. These metabolites enhance the environmental adaptability of plants (Wu et al., 2021). T The enrichment of the Linoleic acid metabolism pathway is potentially associated with UV radiation in Yunnan, which may contribute to the plant’s environmental adaptation (Nie et al., 2024).

C5-branched dibasic acid metabolism is an important metabolic pathway in plants, which is mainly involved in stress response, growth regulation and complex regulation of metabolic network. This metabolic pathway involves several key metabolites, such as α - ketoglutarate and branched chain amino acid degradation products, which can provide energy and regulatory molecules for plants (Kumari and Parida, 2018). Chenopodium quinoa roots significantly increased C5-branched dicarboxylic acid metabolism under waterlogging stress, and regulated urea cycle, starch and sucrose metabolism to adapt to hypoxic environment (Guo et al., 2024). Wheat (Triticum aestivum)significantly increased the metabolism of C5 branched chain dicarboxylic acid and the utilization efficiency of mineral nutrients under the condition of nitrogen and zinc supply (Nie et al., 2023). Therefore, the enriched in C5-branched dicarboxylic acid metabolism pathway may be related to the heavy rainfall in Indonesia and volcanic ash soil.

### 3.4 Redundancy analysis of origin, metabolites and environment of CTLs

**Figure 6:**
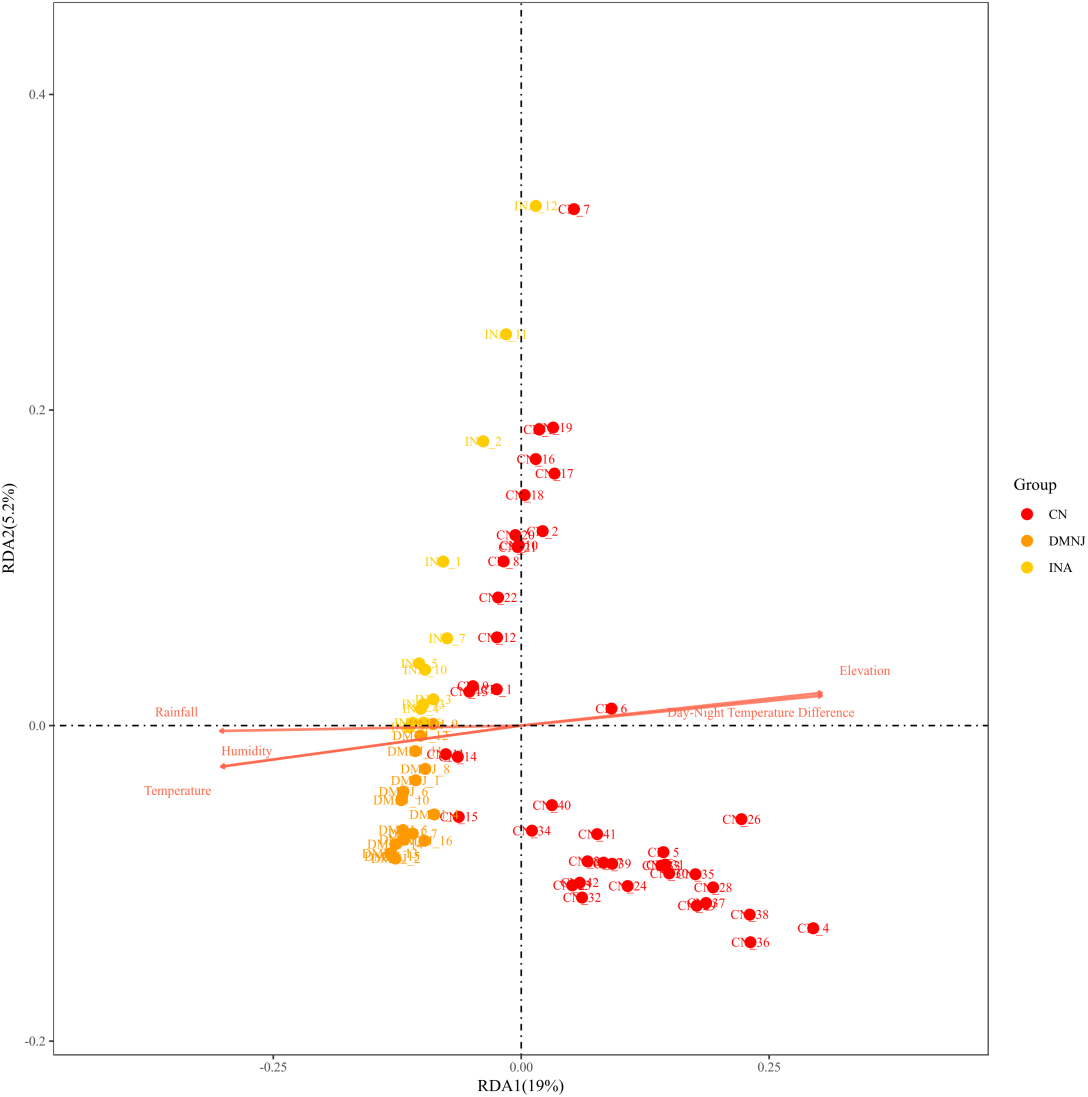
Redundancy analysis (RDA) plots of metabolite and environmental factors of CTLs form Dominica, Indonesia and China (Yunnan).

In order to clarify the correlation characteristics between the metabolites composition of cigars and environmental factors, the data of metabolites from three origins and environmental factors (altitude, temperature difference between day and night, humidity, etc.) were subjected to redundant analysis (RDA), and the results are shown in **Figure 6**.

In RDA ordination chart, RDA1 axis explains 19% of metabolomic variation, which is attributable to the sampled environment, and RDA2 axis explains 5.2% of the variation, with a cumulative explanatory rate of 24.2%, suggesting that environmental factors appear to be significant contributors associated with variation of metabolites in cigars. However, Approximately 75% of the variations remain unexplained by the current set of environmental factors, suggesting the importance of factors such as soil physical and chemical properties, agricultural production, post-harvest processing, varieties, and processes. These will be studied systematically in the future work.

The samples in CN group are concentrated on the right side of RDA1 axis, and their metabolites are mainly driven by altitude and temperature difference between day and night. The samples in DMNJ and INA groups gathered on the left side of RDA1 axis, and the metabolite composition was more regulated by humidity and other factors.

The Elevation and Day–Night Temperature Difference arrows point positively towards RDA1, which is a key gradient distinguishing Yunnan CTLs from Dominica/Indonesia. Yunnan CTLs is located in the quadrant where the two arrows point in the same direction, indicating that its metabolic profile covaries with high elevation and large day-night temperature differences. The Temperature and Humidity arrows are roughly along the negative direction of RDA1 or close to the negative direction of RDA2, indicating that Dominica/Indonesia is more affected by higher temperature/higher humidity conditions.

These results indicated that the heterogeneity of the origin environment is closely associated with the differences in metabolite composition of cigars from different origins, suggesting it may be a key contributing factor.

While our RDA and metabolomics profiling reveal strong links between environmental variables and metabolite accumulation, it is important to emphasize that these results reflect statistical associations rather than direct causal evidence. The observed metabolic patterns co-vary with climatic factors like UV radiation and temperature, but further controlled physiological experiments are required to establish the specific causal mechanisms involved.

### 3.5 Origin traceability

After systematically analyzing the metabonomic characteristics and pathways of CTLs in Dominica, Indonesia and Yunnan, we began to develop origin traceability models by selecting metabolites and classification algorithms that can be used to distinguish CTLs from different countries and cities. At present, it isn’t easy to trace the origin, especially in small regional scale origins. To achieve the traceability of CTLs from different regions at different regional scales, we need to select the appropriate key metabolic indices and classification algorithms.

#### 3.5.1 Screening of metabolic indices

Multivariable methods with unbiased variable selection in the R package (MUVR) are widely used in the screening of biological indicators of metabolic diseases (Shen et al., 2021; Wang et al., 2023) and have been proven to be very effective. We randomly selected 50 samples as the training set and the remaining as the validation set. To improve the classification performance and avoid over fitting and false positives, we use MUVR algorithm based on random forest for multivariate modeling, which can identify Min-, Mid-, and Max-optimal respectively, and perform regression analysis with all-relevant variables.

**Figure 7:**
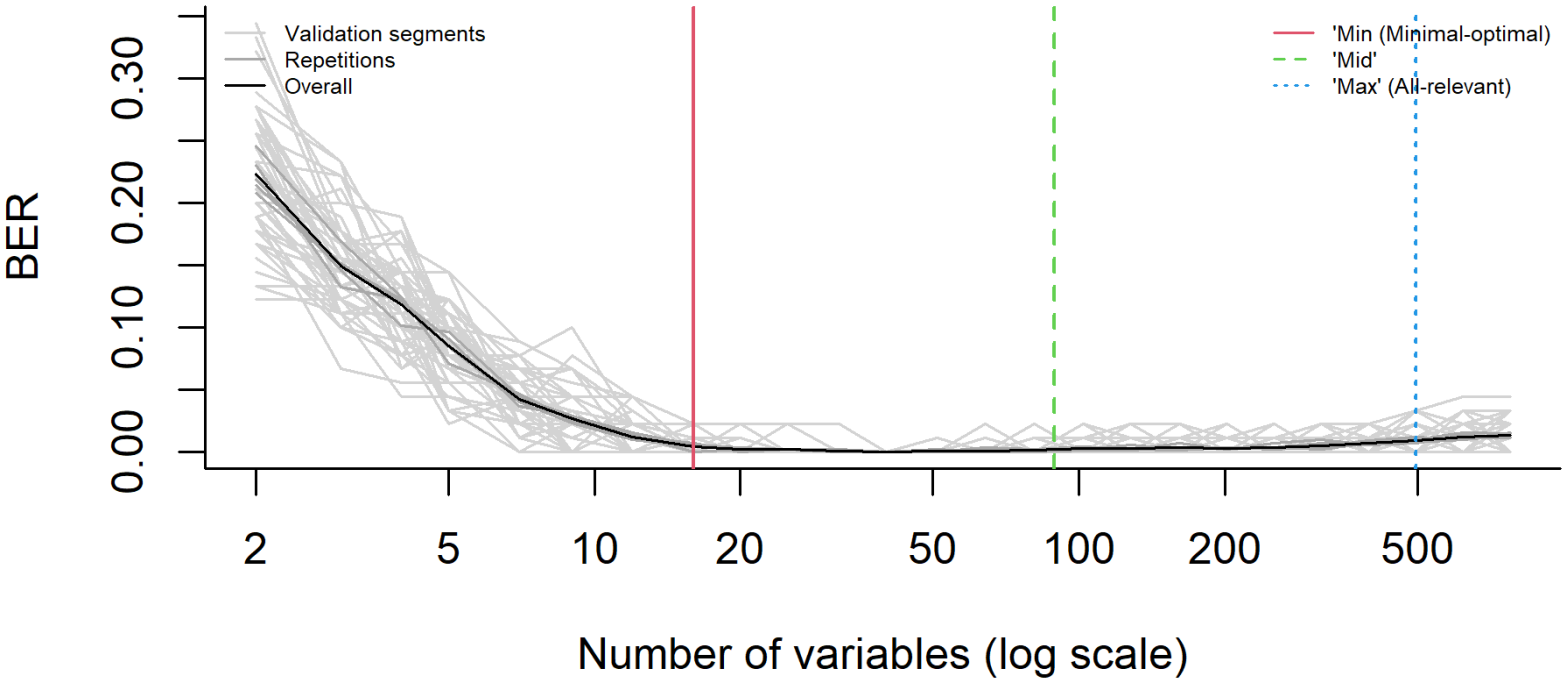
Variable selection based on a multivariate methods with unbiased variable selection in R (MUVR) algorithm. [Balanced Error Rate (BER)].

12 Min-optimal, 77 Mid-optimal, and 497 Max-optimal were screened through the MUVR model, and the corresponding number of error classifications per 100 times were 1, 0, and 1, respectively, show in **Supplementary Table S3** and **Figure 7**. The twelve Min-optimal are the selected metabolic index, they are: Acetylpseudotropine, 3-Methoxysalicylic acid, Monobutyl phthalate, Sinapyl alcohol, Phytyl phosphate, 3-Hydroxyanthranilic acid, 1-Acetylaspidoalbidine, Anacardic acid, Eicosapentaenoic acid ethyl ester, 8-Amino-7-oxononanoate, 2-Amino-3-methoxybenzoic acid, 4-Dodecylbenzenesulfonic acid.

To evaluate the robustness of the 12 index for origin, one-way analysis of variance (ANOVA) Tukey’s test were employed to calculate the differences in index influenced by years and varieties. The P-value results are shown in **Supplementary Table S4**. As indicated in the table, for years, only 8-Amino-7-oxononanoate and 4-Dodecylbenzenesulfonic acid P-values are less than 0.05 (0.045 and 0.026, respectively), indicating that these two indicators were influenced by the years. Meanwhile, for varieties, only Anacardic acid, 2-Amino-3-methoxybenzoic acid, and 4-Dodecylbenzenesulfonic acid P-values are less than 0.05 (0.045 and 0.026, respectively), suggesting that these three indicators were affected by the varieties. Thus, a small part of 12 biomarkers were influenced by both year and cultivar, but most of them remained robust, demonstrating that they are primarily influenced by the geographical origin.

#### 3.5.2 Selection of algorithm

Based on the above metabolic indices, the commonly used classification algorithms of Linear Discriminant Analysis (LDA), Logistic regression (LR), Gaussian Mixture Module (GMM), K-Nearest Neighbors (KNN), Classification Tree (CT), Artificial Neural Network (ANN) and Support Vector Machine (SVM) are selected for comparison. For 71 samples, 50 CTLs were randomly selected as the training set, and the remaining 21 samples were used as the test set for verification. Then the R software was used for 100 repeated modeling tests. The purpose of using random sampling and 100 repetitions is to ensure that all samples are assigned to the training set and test set with the same probability and frequency.

**Figure 8:**
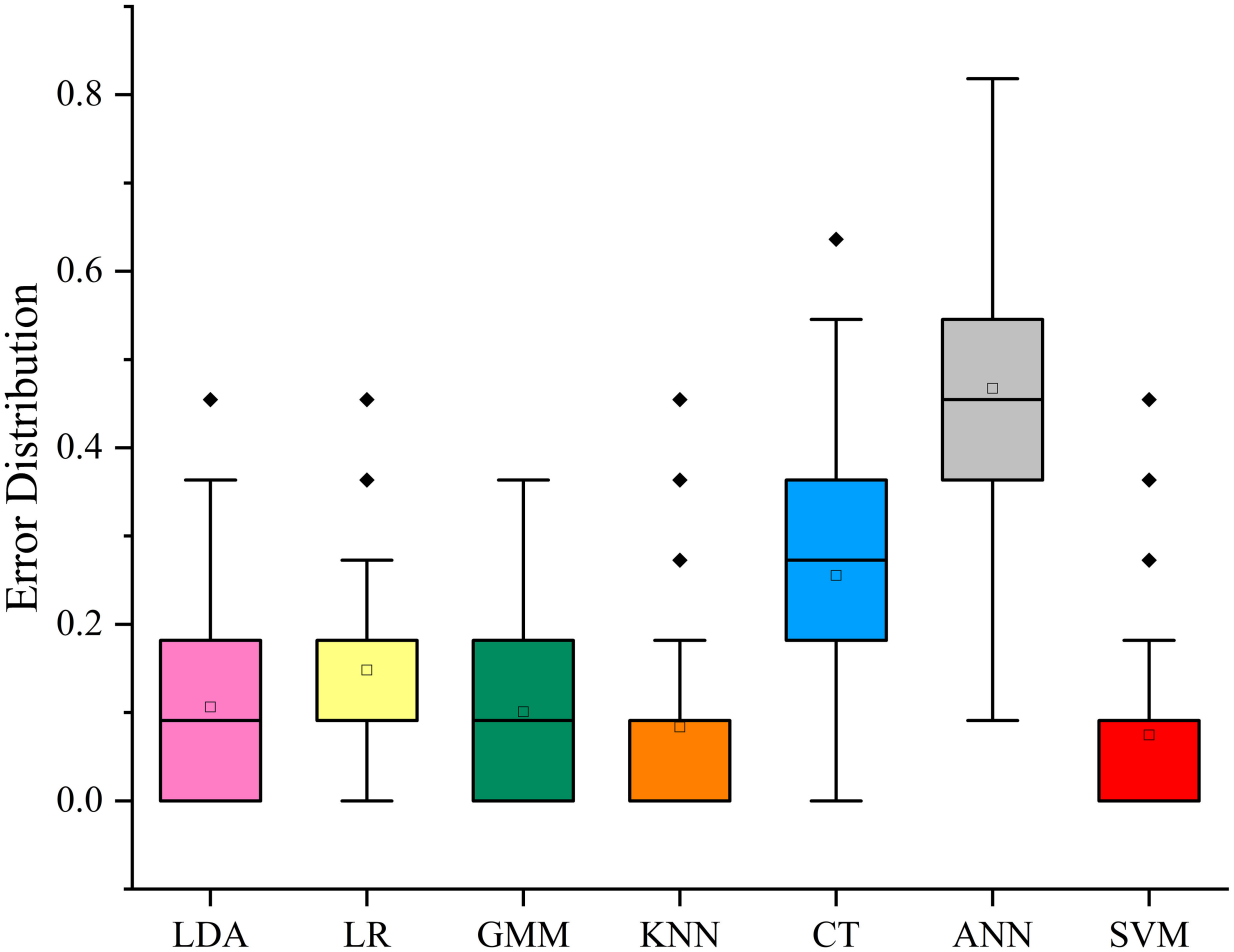
100 prediction errors of SVM, LDA, LR, GMM, and KNN models for different origins CTLs.

The result of the error classification rate is shown in **Figure 8**. The results show that LDA, LR, GMM, KNN and SVM have better classification effect, and the median of 100 false classification rate is about 0.1. The error classification rates of CT and ANN are relatively high, 0.29 and 0.42, respectively. This may be because CT and ANN are suitable for large and complex sample analysis, and small sample size classification is easy to overfit.

Receiver Operating Characteristic Curve (ROC) is a commonly used method to evaluate the classification effect of algorithm model (Obuchowski and Bullen, 2018). Area under curve (AUC) is defined as the area enclosed by the coordinate axis under the ROC curve (Obuchowski and Bullen, 2018). The closer the AUC is to 1, the better the classification effect of the model. The algorithm was further evaluated, and the Hand-Till multiple class classifications ROC curves (Fawcett, 2006; Hand and Till, 2001) were constructed by using LDA, LR, GMM, KNN and SVM, respectively. The results are shown in **Supplementary Figure S2**. It can be seen that LDA, LR, GMM, KNN and SVM have a good classification effect in CTLs origin identification, with their AUC near 1. The results are consistent with the previous 100 false classification rate results, which once again confirm the classification accuracy of these algorithms. The indices obtained based on MUVR screening method can accurately trace the origin, whether in large national scale or in small prefecture scale that is difficult to trace, proving that MUVR is robust in the screening of geo-origin characteristic index.

## 4 Conclusions

In this paper, the CTLs from Dominica, Indonesia and Yunnan have been studied by non-targeted omics. The metabolic pathways of different metabolites in CTLs from Yunnan, Dominica and Indonesia were enriched, and six metabolic pathways such as Flavone and flavonol biosynthesis, Phenylpropanoid biosynthesis and Nicotinate and nicotinamide metabolism, Betalain biosynthesis, Linoleic acid metabolism, C5-Branched dibasic acid metabolism were obtained. This may be related to the geographical and climatic characteristics of Yunnan, such as UV light intensity, relative dryness (drought), large temperature difference between day and night, and may also be related to the fermentation process. The content of polyphenol flavor compounds in Yunnan CTLs is high, which may be one of the material basis of the special flavor of Yunnan cigars. The content of polyphenols was related to the intensity of UV-light, so that the flavor characteristics of Yunnan CTLs had a certain relationship with the high UV radiation in Yunnan. This study provides a comprehensive map of the metabolic differences in CTLs across different origins. However, the current study design is associative; therefore, while the geographical climate appears to shape the metabolic profile, future research employing controlled environmental chambers is necessary to confirm these causal relationships.

Based on hundreds of metabolites obtained from non-targeted metabolism analysis of CTLs, 12 characteristic metabolites related to origin were screened by MUVR method. Through the multiple algorithms modeling and verification analysis of sample training set-test set, it is found that LDA, LR, GMM, KNN and SVM have a very good effect on the origin traceability of CTLs, with the median of 100 false classification rate about 0.1 and the AUC close to 1. MUVR index screening combined with some algorithms, has achieved accurate traceability of CTLs’ origins at different regional scales, can accurately trace the origin of CTLs at different scales, whether at the national level or the more challenging regional level.

This study provides valuable insights into the relationship between the geographical environment of Yunnan and the metabolic characteristics of CTLs, and offers a robust and efficient method for origin tracing of CTLs. It has practical significance for the agricultural production and quality authentication of Yunnan CTLs.

## Data Availability

The original contributions presented in the study are included in the article/**Supplementary Material**. Further inquiries can be directed to the corresponding author/s.
