# Supplementary material for: Metabolomic analysis of Yunnan cigar tobacco leaves: impact of geography and climate on flavor characteristics and machine learning-based origin traceability
Source: Front Plant Sci. 2026 Feb 18;16:1703429. doi: 10.3389/fpls.2025.1703429 (PMC12957283; doi:10.3389/fpls.2025.1703429)
Supplement: Supplementary file 1 [file Supplementaryfile1.doc]

**Metabolomic Analysis of Yunnan Cigar Tobacco Leaves: Impact of Geography and Climate on Flavor Characteristics and Machine Learning-Based Origin Traceability**

Yuping Wu^1†^, Guijuan Zhao^2,3†^, Yi Li ^2^, Guifeng Li ^2^, Wenyuan Wang^3^, Lei Yang^3^, Zhonglong Lin^2^, Heng Yao^1^, Fangchan Jiao^1^, Gaokun Zhao^1 *^, Yongping Li ^1^, Guanghai Zhang^1^, Meiwei Zhao^4 *^, Tao Zhang^3 *^ and Jin Wang^3 *^

^1^Yunnan Academy of Tobacco Agricultural Sciences, Kunming, China,

^2^Key Laboratory of Natural Products Synthetic Biology of Ethnic Medicina Endophytes, Yunnan Minzu University, Kunming, China,

^3^Yunnan Key Laboratory of Tobacco Chemistry, China Tobacco Yunnan Industrial Co., Ltd., Kunming, China,

^4^College of Agronomy, Yunnan Urban Agricultural Engineering and Technological Research Center, Kunming University, Kunming, China

^‡^Both are the first authors.


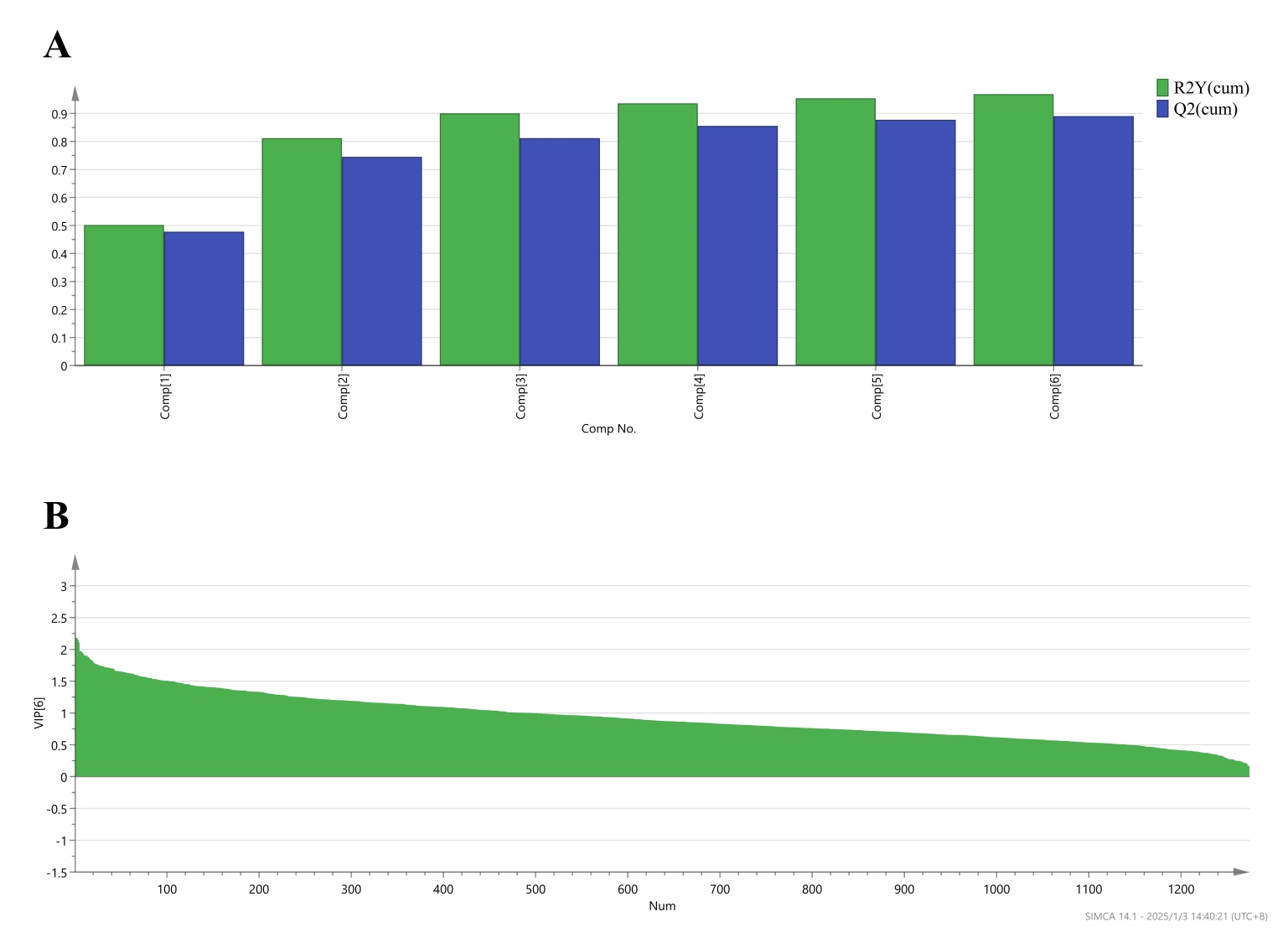


Fig. S1 The summary fit (A) and VIP (B) of PLS-DA of CTLs form different regions.


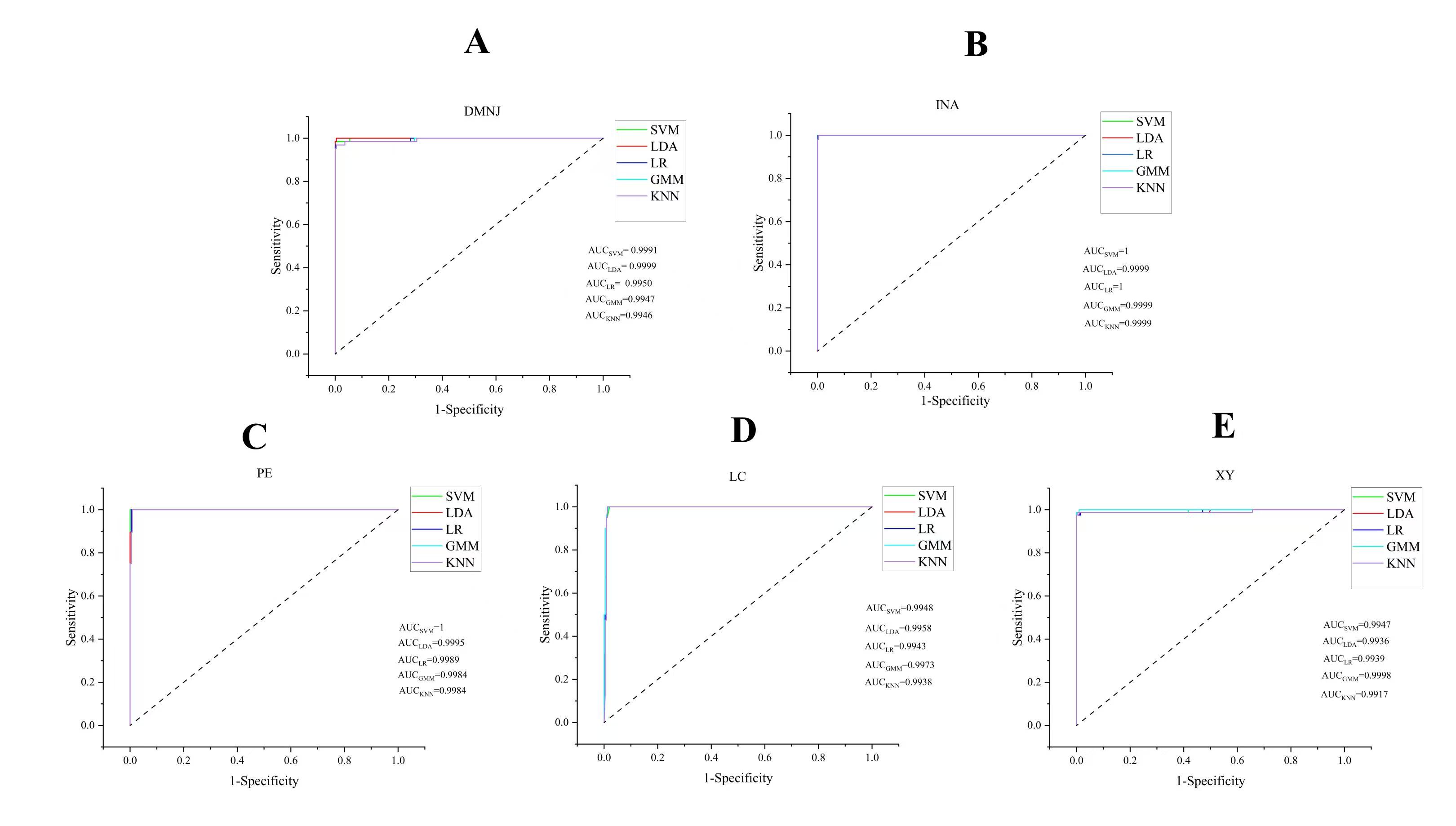


Figure S2. ROC curves of external validation of SVM, LDA, LR, GMM, and KNN models for different origins CTLs

Table S1. Significantly different metabolites in Dominican vs Yunnan CTLs.

| **Index** | **Class I** | **VIP** | **P-value** | **FDR** | **Fold_Change** | **Log2FC** | **Type** |
| --- | --- | --- | --- | --- | --- | --- | --- |
| Caulophylline | Alkaloids | 2.098548 | 0.00652 | 0.021782 | 4.37697 | 2.129932 | up |
| Kaempferol-3-O-rutinoside | Flavonoids | 1.380461 | 1.68E-05 | 0.000219 | 0.179658 | -2.47667 | down |
| Homovanillic acid | Phenols and derivatives | 1.101506 | 0.000418 | 0.002428 | 0.274866 | -1.8632 | down |
| Caffeic acid | Polyketides[PK] | 1.24386 | 0.000411 | 0.002428 | 0.160792 | -2.63674 | down |
| Gallic acid | Benzene and derivatives | 1.478714 | 0.000418 | 0.002428 | 0.11164 | -3.16308 | down |
| Hydroxygenkwanin | Flavonoids | 1.581404 | 1.34E-05 | 0.000186 | 0.285988 | -1.80597 | down |
| Isoquercitrin | Flavonoids | 1.172258 | 0.001604 | 0.006973 | 0.198881 | -2.33002 | down |
| Astragalin | Flavonoids | 1.279572 | 2.22E-05 | 0.000266 | 0.177399 | -2.49493 | down |
| 3-Methoxy-5,7,3',4'-tetrahydroxy-flavone | Flavonoids | 1.856981 | 2.34E-07 | 1.22E-05 | 0.109856 | -3.18632 | down |
| Isokaempferide | Flavonoids | 1.620131 | 4.90E-06 | 0.000106 | 0.265615 | -1.91259 | down |
| Parthenolide | Terpenoids | 1.499068 | 1.15E-06 | 3.90E-05 | 0.217486 | -2.201 | down |
| Cytisine | Alkaloids | 1.499278 | 0.006637 | 0.022067 | 2.161508 | 1.112038 | up |
| 4-Heptylphenol | Polyketides[PK] | 2.349107 | 4.33E-10 | 1.12E-07 | 0.092153 | -3.43983 | down |
| L-Histidine | Amino acids | 1.355768 | 0.013232 | 0.037277 | 0.358949 | -1.47815 | down |
| Quinolinic acid | Pyridine and derivatives | 1.784489 | 0.010711 | 0.032151 | 3.796271 | 1.924583 | up |
| Skatole | Indoles | 1.624762 | 1.04E-06 | 3.68E-05 | 0.406603 | -1.29831 | down |
| D-(+)-Arabitol | Carbohydrates | 1.11969 | 0.000502 | 0.002659 | 0.312803 | -1.67667 | down |
| Dodecanedioic acid | Fatty acyls[FA] | 2.129585 | 8.98E-06 | 0.000149 | 2.401954 | 1.264208 | up |
| Indole-3-lactic acid | Indole and derivatives | 1.367222 | 2.87E-05 | 0.000315 | 0.358763 | -1.4789 | down |
| Hexadecanedioic acid | Fatty acyls[FA] | 2.104101 | 0.000469 | 0.002577 | 3.883396 | 1.957319 | up |
| trans-3-Indoleacrylic acid | Indoles | 1.627821 | 6.21E-06 | 0.000116 | 0.330681 | -1.59649 | down |
| Hydrocinnamic acid | Phenylpropanoic acids | 1.794238 | 3.21E-07 | 1.47E-05 | 2.345406 | 1.229837 | up |
| Xanthurenic acid | Quinoline carboxylic acids | 2.172833 | 7.80E-06 | 0.000135 | 2.801683 | 1.486294 | up |
| L-Tryptophan | Amino acids | 1.208259 | 0.000212 | 0.001463 | 0.201421 | -2.31171 | down |
| trans-Cinnamic acid | Phenylpropanoids | 1.580297 | 0.000184 | 0.001304 | 2.023769 | 1.017044 | up |
| Ferulic acid | Phenylpropanoids | 1.309279 | 9.49E-07 | 3.51E-05 | 0.339037 | -1.56048 | down |
| 3-Hydroxyanthranilic acid | Benzene and derivatives | 1.343284 | 0.001086 | 0.005151 | 2.180849 | 1.12489 | up |
| Benzoylformic acid | Benzene and derivatives | 1.516026 | 3.46E-06 | 7.70E-05 | 0.321325 | -1.6379 | down |
| L-Norleucine | Fatty acyls[FA] | 1.119322 | 0.000727 | 0.003604 | 0.481244 | -1.05516 | down |
| Phthalic acid | Benzene and derivatives | 1.892642 | 5.43E-07 | 2.22E-05 | 0.187941 | -2.41165 | down |
| Capsaicin | Amino acid related compounds | 1.43768 | 0.001956 | 0.008314 | 0.269362 | -1.89238 | down |
| 3-Indolepropionic acid | Indole and derivatives | 1.567701 | 1.98E-05 | 0.000241 | 0.499185 | -1.00235 | down |
| N-Methylnicotinamide | Pyridine and derivatives | 1.813441 | 0.009736 | 0.029359 | 3.785383 | 1.920439 | up |
| Palmitoleic acid | Fatty acyls[FA] | 1.943308 | 0.001531 | 0.006692 | 2.873692 | 1.522905 | up |
| Solanidine | Alkaloids | 1.641113 | 0.003018 | 0.012165 | 3.259167 | 1.704503 | up |
| Rutin | Flavonoids | 1.260979 | 0.000204 | 0.001419 | 0.182628 | -2.45302 | down |
| 2-Amino-1,3,4-octadecanetriol | Sphingolipids[SP] | 1.932947 | 3.15E-06 | 7.42E-05 | 0.442062 | -1.17768 | down |
| Quercetin | Flavonoids | 1.360147 | 0.005534 | 0.019518 | 0.099614 | -3.32751 | down |
| Kaempferol | Flavonoids | 1.495704 | 8.87E-06 | 0.000149 | 0.168761 | -2.56694 | down |
| Oleoyl-L-a-lysophosphatidic acid | Glycerophospholipids[GP] | 1.589551 | 0.012818 | 0.036263 | 0.078876 | -3.66427 | down |
| Monobutyl phthalate | Benzene and derivatives | 1.966132 | 1.25E-05 | 0.00018 | 0.100714 | -3.31166 | down |
| Mono(2-ethylhexyl) phthalate (MEHP) | Benzene and derivatives | 1.610389 | 0.001122 | 0.00526 | 0.312577 | -1.67772 | down |
| Trihexyphenidyl | Amines | 1.744193 | 3.02E-05 | 0.000317 | 2.235053 | 1.160309 | up |
| DL-Tryptophan | Indole and derivatives | 1.702667 | 1.86E-06 | 5.10E-05 | 0.27007 | -1.88859 | down |
| Neohesperidin | Flavonoids | 1.277911 | 0.00066 | 0.003333 | 0.243432 | -2.03841 | down |
| Citreoviridin | Fungal toxins | 1.381373 | 0.000124 | 0.000952 | 0.421065 | -1.24789 | down |
| 4-Ethylbenzaldehyde | Benzene and derivatives | 1.880769 | 1.48E-10 | 5.78E-08 | 0.454011 | -1.1392 | down |
| 2-Anisic acid | Benzene and derivatives | 1.276836 | 0.001931 | 0.008255 | 2.003289 | 1.002371 | up |
| Sinapinic acid | Phenylpropanoids | 1.105195 | 0.000555 | 0.002877 | 0.203428 | -2.29741 | down |
| a-Zearalanol | Fungal toxins | 1.156145 | 3.65E-05 | 0.000355 | 0.26532 | -1.9142 | down |
| Harmane | Alkaloids | 1.134526 | 0.001413 | 0.006281 | 2.619862 | 1.389491 | up |
| Matairesinol | Lignans | 1.571931 | 9.54E-05 | 0.000789 | 0.337381 | -1.56755 | down |
| Ethoxyquin | Quinolones and derivatives | 1.593479 | 1.67E-06 | 5.00E-05 | 0.214836 | -2.21869 | down |
| 3-Hydroxybenzyl alcohol | Alcohols | 1.297724 | 0.01525 | 0.041484 | 0.07791 | -3.68205 | down |
| Dihydroresveratrol | Polyketides[PK] | 1.59169 | 0.000123 | 0.000952 | 0.186816 | -2.42031 | down |
| Ellipticine | Alkaloids | 1.489801 | 1.93E-05 | 0.000239 | 2.163412 | 1.113308 | up |
| N-Arachidonoyl dopamine | 0 | 1.955002 | 3.24E-08 | 2.80E-06 | 0.259759 | -1.94476 | down |
| Glycodeoxycholic acid | Sterol lipids[ST] | 1.189496 | 0.004598 | 0.016715 | 2.410491 | 1.269327 | up |
| Glycoursodeoxycholic acid | Bile acids, alcohols and derivatives | 1.261029 | 0.009201 | 0.028293 | 2.574627 | 1.364364 | up |
| 7-Methylguanine | Purines and derivatives | 1.855816 | 0.000106 | 0.000839 | 3.327918 | 1.73462 | up |
| Melatonin | Hormones and transmitters | 1.471246 | 4.78E-05 | 0.000438 | 0.394995 | -1.3401 | down |
| D-(+)-Glucosamine | Carbohydrates | 1.355558 | 0.008564 | 0.026976 | 0.148165 | -2.75473 | down |
| 3-Aminosalicylic acid | Benzene and derivatives | 1.357536 | 0.012713 | 0.036231 | 0.132706 | -2.9137 | down |
| Polydatin | Polyketides[PK] | 1.29101 | 8.95E-07 | 3.48E-05 | 0.48278 | -1.05056 | down |
| Stevioside | Terpenoids | 1.902324 | 5.52E-05 | 0.000487 | 0.235097 | -2.08867 | down |
| Dihydrocoumarin | Coumarins and derivatives | 1.074032 | 0.014314 | 0.039489 | 0.475796 | -1.07159 | down |
| Bilobalide | Terpenoids | 1.619865 | 1.90E-06 | 5.10E-05 | 0.238667 | -2.06693 | down |
| 4-Hydroperoxy-2-nonenal | Carbonyl compounds | 1.353438 | 5.57E-05 | 0.000487 | 0.49403 | -1.01733 | down |
| 2-Amino-3-methoxybenzoic acid | Benzene and derivatives | 1.334129 | 0.008217 | 0.026172 | 2.364977 | 1.241826 | up |
| Dihydrobiopterin | Pteridines and derivatives | 1.875718 | 4.39E-08 | 3.42E-06 | 0.280194 | -1.8355 | down |
| N6-(L-1,3-Dicarboxypropyl)-L-lysine | Amino acids, peptides, and analogues | 1.135617 | 0.005166 | 0.018521 | 0.461942 | -1.11422 | down |
| sn-Glycero-3-phosphocholine | Glycerophospholipids[GP] | 1.690508 | 0.000159 | 0.001171 | 2.298389 | 1.200623 | up |
| 3-Methyladenine | Purines and derivatives | 1.67547 | 5.06E-07 | 2.19E-05 | 0.407025 | -1.29681 | down |
| N-(6-Aminohexanoyl)-6-aminohexanoate | Fatty acyls[FA] | 1.283618 | 0.002168 | 0.00907 | 0.345234 | -1.53435 | down |
| Benzoin 2-Hydroxy-1,2-diphenylethanone | Polyketides[PK] | 1.590762 | 0.000123 | 0.000952 | 0.187212 | -2.41725 | down |
| Luteolin | Flavonoids | 1.142708 | 0.000431 | 0.002469 | 0.313141 | -1.67511 | down |
| Xylobiose | Carbohydrates | 1.512784 | 0.007692 | 0.024936 | 0.194927 | -2.359 | down |
| Orsellinate o-Orsellinic acid | Polyketides[PK] | 1.27707 | 0.006052 | 0.021114 | 3.413866 | 1.771407 | up |
| Tolylacetate p-Cresyl acetate | Benzene and derivatives | 1.148247 | 0.002905 | 0.011773 | 0.496742 | -1.00943 | down |
| Phylloquinone | Vitamins | 1.865411 | 3.72E-05 | 0.000357 | 2.637677 | 1.399268 | up |
| Sinapyl alcohol | Phenylpropanoids | 2.34015 | 1.48E-05 | 0.000198 | 4.056862 | 2.020364 | up |
| Isoorientin 2''-O-rhamnoside | Flavonoids | 1.178533 | 5.64E-06 | 0.000113 | 0.24129 | -2.05116 | down |
| Isocorypalmine | Alkaloids | 1.667363 | 3.30E-05 | 0.000338 | 2.422831 | 1.276694 | up |
| 13-(2-Methylcrotonoyl)oxylupanine | Alkaloids | 1.606169 | 5.57E-05 | 0.000487 | 2.408925 | 1.26839 | up |
| (3S,4S)-3-Hydroxytetradecane-1,3,4-tricarboxylate | Fatty acyls[FA] | 1.726833 | 0.000135 | 0.00103 | 4.628275 | 2.210474 | up |
| 10-Hydroxydihydrosanguinarine | Alkaloids | 1.365661 | 0.004549 | 0.016694 | 2.677186 | 1.420717 | up |
| Elymoclavine | Alkaloids | 1.62245 | 0.000347 | 0.002156 | 3.232665 | 1.692724 | up |
| Deoxyloganin | Terpenoids | 1.455615 | 0.012006 | 0.034982 | 3.976857 | 1.991629 | up |
| Fusidic acid | Sterol lipids[ST] | 1.361885 | 1.72E-05 | 0.000219 | 0.473003 | -1.08008 | down |
| 3-Hydroxyquinine | Alkaloids | 1.733524 | 7.98E-08 | 5.65E-06 | 0.236575 | -2.07963 | down |
| Karakoline | Alkaloids | 2.274985 | 1.12E-05 | 0.000171 | 4.914671 | 2.297095 | up |
| Nudicauline | Alkaloids | 1.587647 | 0.003437 | 0.013557 | 3.239941 | 1.695967 | up |
| 6-Acetylpicropolin | Terpenoids | 1.256528 | 0.003374 | 0.013392 | 2.468221 | 1.303472 | up |
| Carnosol | Terpenoids | 1.859507 | 0.013683 | 0.038018 | 5.071678 | 2.342463 | up |
| Canthin-6-one 6H-Indolo(3,2,1-de)(1,5)naphthyridin-6-one | Alkaloids and derivatives | 2.172789 | 0.000266 | 0.001742 | 3.350935 | 1.744564 | up |
| (-)-Quebrachamine | Alkaloids and derivatives | 1.88457 | 1.20E-09 | 2.34E-07 | 0.163896 | -2.60915 | down |
| Decursin | Coumarins | 1.50742 | 0.008062 | 0.025986 | 2.486716 | 1.314242 | up |
| Arctiopicrin | Terpenoids | 1.225984 | 0.00145 | 0.006373 | 0.355833 | -1.49073 | down |
| (-)-Annonaine Anonaine | Alkaloids | 1.126339 | 0.000417 | 0.002428 | 0.433509 | -1.20587 | down |
| Capnoidine (-)-Adlumidine | Alkaloids | 1.304822 | 0.002087 | 0.008775 | 2.78376 | 1.477035 | up |
| (+-)-Carnegine Carnegine | Alkaloids | 1.509761 | 0.00881 | 0.027306 | 3.271987 | 1.710167 | up |
| Cassythine Cassyfiline | Alkaloids | 1.635784 | 1.45E-05 | 0.000198 | 0.248969 | -2.00596 | down |
| Fetidine | Alkaloids | 1.547897 | 2.69E-05 | 0.000303 | 0.379775 | -1.39678 | down |
| Hymenoxon | Terpenoids | 1.500637 | 1.04E-05 | 0.000164 | 0.276285 | -1.85577 | down |
| Laurenobiolide | Terpenoids | 1.425561 | 9.73E-05 | 0.000796 | 0.197314 | -2.34143 | down |
| Linifolin A | Terpenoids | 1.318578 | 0.004326 | 0.016026 | 2.228352 | 1.155977 | up |
| Orizabin | Terpenoids | 1.356962 | 0.000351 | 0.002156 | 0.35625 | -1.48904 | down |
| Scorpioidin | Terpenoids | 1.254828 | 5.06E-05 | 0.000458 | 0.305378 | -1.71133 | down |
| Laudanosine | Alkaloids | 1.227215 | 0.000502 | 0.002659 | 0.263558 | -1.92381 | down |
| Vernoflexuoside | Terpenoids | 1.128237 | 9.30E-05 | 0.000778 | 0.181252 | -2.46393 | down |
| Boschnialactone | Terpenoids | 1.704179 | 1.33E-07 | 7.93E-06 | 0.151937 | -2.71845 | down |
| Fisetin 8-C-glucoside | Polyketides[PK] | 1.131955 | 0.000576 | 0.002948 | 0.210822 | -2.2459 | down |
| Athyriol 3-Methoxy-1,6,7-trihydroxyxanthone | Polyketides[PK] | 1.50606 | 0.001228 | 0.005665 | 2.175917 | 1.121624 | up |
| Ammodendrine | Alkaloids | 1.589361 | 0.012381 | 0.035544 | 5.826407 | 2.542607 | up |
| Isolobinine | Alkaloids | 1.515679 | 0.000481 | 0.002597 | 2.249749 | 1.169764 | up |
| Feruloylputrescine | Polyketides[PK] | 1.06505 | 0.01276 | 0.036231 | 2.246376 | 1.1676 | up |
| (+)-Elaeocarpine | Alkaloids | 1.487351 | 2.71E-06 | 6.80E-05 | 0.310148 | -1.68897 | down |
| Elaeokanine C | Alkaloids | 1.363085 | 0.00026 | 0.001717 | 2.76535 | 1.467462 | up |
| Arborinine | Alkaloids | 1.595662 | 0.00648 | 0.021782 | 4.244328 | 2.085536 | up |
| Atalaphylline | Alkaloids | 1.128538 | 0.000692 | 0.003449 | 0.484503 | -1.04542 | down |
| Dictamnine | Alkaloids | 1.489271 | 9.75E-08 | 6.32E-06 | 2.186121 | 1.128373 | up |
| 4'-Hydroxy-3'-prenylacetophenone | Carbonyl compounds | 1.582233 | 1.45E-06 | 4.50E-05 | 0.301229 | -1.73107 | down |
| Lemobiline | Alkaloids | 1.179966 | 0.000248 | 0.001648 | 0.322917 | -1.63076 | down |
| Robustine | Alkaloids | 1.441541 | 3.23E-05 | 0.000335 | 0.259392 | -1.94679 | down |
| Rutacridone epoxide | Alkaloids | 1.527778 | 5.31E-06 | 0.000112 | 2.878454 | 1.525294 | up |
| Cinegalline | Alkaloids | 1.052253 | 0.000571 | 0.002942 | 0.236462 | -2.08032 | down |
| 5-(Heptadec-12-enyl)resorcinol | Polyketides[PK] | 2.221588 | 1.70E-05 | 0.000219 | 4.828601 | 2.271605 | up |
| Meteloidine | Alkaloids | 1.240651 | 0.01705 | 0.045585 | 2.120645 | 1.084503 | up |
| Strobamine | Alkaloids | 2.035459 | 3.05E-06 | 7.41E-05 | 3.86484 | 1.950409 | up |
| Benalaxyl | Amino acids, peptides, and analogues | 1.720797 | 0.000223 | 0.001524 | 3.072604 | 1.619462 | up |
| 6alpha-Glucuronosylhyodeoxycholate | Sterol lipids[ST] | 1.386219 | 0.01233 | 0.035528 | 2.602754 | 1.380039 | up |
| 7-Epiloganin tetraacetate | Prenol lipids[PR] | 1.361465 | 5.66E-06 | 0.000113 | 0.245975 | -2.02342 | down |
| alpha-Ionone | Terpenoids | 2.309726 | 3.85E-09 | 4.99E-07 | 0.168211 | -2.57166 | down |
| Acetylpseudotropine | Alkaloids | 2.33601 | 1.16E-05 | 0.000174 | 5.461682 | 2.449345 | up |
| 2-Phytyl-1,4-naphthoquinone | Prenol lipids[PR] | 1.376307 | 8.15E-05 | 0.000689 | 0.192425 | -2.37763 | down |
| 4-Propylphenol | Benzene and derivatives | 2.232433 | 2.06E-09 | 3.20E-07 | 0.092934 | -3.42765 | down |
| p-Methoxystilbene | Polyketides[PK] | 1.23862 | 0.010786 | 0.032151 | 2.356015 | 1.236349 | up |
| 4,5-Dihydro-4-hydroxy-5-S-glutathionyl-benzo[a]pyrene | Amino acids, peptides, and analogues | 1.311539 | 0.008083 | 0.025986 | 0.334884 | -1.57827 | down |
| Apocholic acid | Sterol lipids[ST] | 1.651302 | 0.004285 | 0.015952 | 2.260747 | 1.176799 | up |
| Chalcone | Polyketides[PK] | 1.411051 | 0.003845 | 0.014735 | 2.849496 | 1.510707 | up |
| 1'-Hydroxy-gamma-carotene | Prenol lipids[PR] | 1.33444 | 0.00017 | 0.00121 | 0.49714 | -1.00828 | down |
| Abscisic acid glucose ester | Prenol lipids[PR] | 1.388936 | 3.37E-06 | 7.70E-05 | 0.360716 | -1.47106 | down |
| (+)-7-Isomethyljasmonate | Fatty acyls[FA] | 1.337271 | 1.76E-06 | 5.06E-05 | 0.474479 | -1.07558 | down |
| Volicitin | Fatty acyls[FA] | 1.433123 | 0.000453 | 0.002536 | 2.128594 | 1.089901 | up |
| Ferrous lactate | Organic acids | 1.324919 | 0.011499 | 0.033888 | 3.43559 | 1.780558 | up |
| Kurarinol | Polyketides[PK] | 1.32224 | 0.012187 | 0.035379 | 2.897891 | 1.535003 | up |
| Capsi-amide | Organic acids | 1.928759 | 0.003265 | 0.013028 | 3.799611 | 1.925852 | up |
| 9beta-Pimara-7,15-diene | Prenol lipids[PR] | 2.016192 | 1.10E-11 | 8.56E-09 | 0.243416 | -2.03851 | down |
| 1,1'-Dihydroxy-1,1',2,2'-tetrahydrolycopene | Prenol lipids[PR] | 1.366658 | 0.000101 | 0.000812 | 0.488076 | -1.03482 | down |
| Iriomoteolide 1a | Marine biotoxins | 1.596097 | 2.08E-08 | 2.02E-06 | 0.303889 | -1.71838 | down |
| Pseudoargiopinin III | Venoms | 2.095385 | 0.000515 | 0.002708 | 3.236075 | 1.694245 | up |
| N-(4-Coumaroyl)-L-homoserine lactone | Fatty acyls[FA] | 1.593049 | 2.93E-05 | 0.000315 | 2.453681 | 1.294948 | up |
| Phytyl phosphate | Prenol lipids[PR] | 2.033857 | 0.000331 | 0.002091 | 3.07813 | 1.622054 | up |
| 4-Hydroxy-3-methylbenzoate | Benzene and derivatives | 1.48893 | 1.74E-07 | 9.69E-06 | 0.211371 | -2.24215 | down |
| (3E)-4,8-Dimethylnona-1,3,7-triene | Terpenoids | 1.833935 | 1.25E-06 | 4.06E-05 | 0.202092 | -2.30691 | down |
| (6Z,9Z)-Hexadecadienoic acid | Fatty acyls[FA] | 1.927696 | 0.002611 | 0.010804 | 3.995178 | 1.99826 | up |

Table S2. Significantly different metabolites in Indonesian vs Yunnan CTLs.

| **Index** | **Class I** | **VIP** | **P-value** | **FDR** | **Fold_Change** | **Log2FC** | **Type** |
| --- | --- | --- | --- | --- | --- | --- | --- |
| Homovanillic acid | Phenols and derivatives | 1.767018 | 0.001425 | 0.004364 | 7.540521 | 2.914664 | up |
| Gallic acid | Benzene and derivatives | 1.350359 | 0.000737 | 0.002606 | 0.139789 | -2.83867 | down |
| Hydroxygenkwanin | Flavonoids | 1.330577 | 1.81E-06 | 2.17E-05 | 0.255235 | -1.9701 | down |
| 3-Methoxyphenylacetic acid | Benzene and derivatives | 1.257385 | 0.001524 | 0.004614 | 2.025103 | 1.017996 | up |
| 3-Methoxy-5,7,3',4'-tetrahydroxy-flavone | Flavonoids | 1.731693 | 9.08E-08 | 1.91E-06 | 0.07259 | -3.78409 | down |
| Isokaempferide | Flavonoids | 1.391773 | 5.58E-07 | 8.04E-06 | 0.226125 | -2.14481 | down |
| Senecionine | Alkaloids | 1.154842 | 0.002724 | 0.007409 | 2.225307 | 1.154004 | up |
| 4-Heptylphenol | Polyketides[PK] | 1.881763 | 1.79E-09 | 1.07E-07 | 0.138968 | -2.84718 | down |
| Gardenin B | Flavonoids | 1.969092 | 0.000244 | 0.000994 | 221.0848 | 7.788456 | up |
| Adenosine 3'5'-cyclic monophosphate | Nucleic acids | 1.575854 | 0.003373 | 0.009019 | 0.104216 | -3.26235 | down |
| Guanine | Nucleic acids | 1.645938 | 2.45E-10 | 2.38E-08 | 0.234192 | -2.09423 | down |
| L-Glutamic acid | Amino acids | 1.686187 | 1.28E-06 | 1.59E-05 | 0.415373 | -1.26752 | down |
| L-Phenylalanine | Amino acids | 1.047181 | 0.000172 | 0.000722 | 0.454412 | -1.13793 | down |
| L-Histidine | Amino acids | 1.728463 | 4.16E-05 | 0.000244 | 0.07212 | -3.79347 | down |
| L-Dopa | Amino acids | 1.989377 | 0.007023 | 0.01676 | 11.9842 | 3.583061 | up |
| Phenylacetic acid | Benzene and derivatives | 1.352058 | 0.004556 | 0.01166 | 2.257212 | 1.174542 | up |
| Quinolinic acid | Pyridine and derivatives | 1.716394 | 0.004252 | 0.011027 | 4.514116 | 2.174443 | up |
| Urocanic acid | Imidazoles | 1.161528 | 1.64E-05 | 0.000122 | 0.251304 | -1.9925 | down |
| 2-Furoylglycine | Amino acids, peptides, and analogues | 1.622598 | 0.007405 | 0.017405 | 7.454357 | 2.898084 | up |
| Hexanoic acid | Organic acids | 1.152767 | 0.000308 | 0.001218 | 2.352784 | 1.234369 | up |
| D-(+)-Arabitol | Carbohydrates | 1.102571 | 0.00012 | 0.000545 | 0.246797 | -2.0186 | down |
| Dodecanedioic acid | Fatty acyls[FA] | 1.877061 | 1.06E-06 | 1.42E-05 | 2.506577 | 1.325719 | up |
| Citraconic acid | Fatty acyls[FA] | 2.224436 | 7.99E-08 | 1.73E-06 | 2.35409 | 1.235169 | up |
| Indole-3-lactic acid | Indole and derivatives | 1.298628 | 3.85E-06 | 3.91E-05 | 0.292445 | -1.77376 | down |
| Hexadecanedioic acid | Fatty acyls[FA] | 1.991041 | 0.001236 | 0.003877 | 4.4844 | 2.164915 | up |
| Linoleic acid | Fatty acyls[FA] | 1.748861 | 4.23E-08 | 1.12E-06 | 0.388731 | -1.36316 | down |
| Hexanoylglycine | Amino acids, peptides, and analogues | 1.810764 | 3.20E-05 | 0.000201 | 2.213336 | 1.146222 | up |
| 2-Hydroxyoctanoic acid | Fatty acyls[FA] | 2.143782 | 2.00E-07 | 3.62E-06 | 3.386319 | 1.759718 | up |
| Pipecolic acid | Alkaloids | 1.279592 | 0.002143 | 0.006086 | 0.295089 | -1.76078 | down |
| trans-3-Indoleacrylic acid | Indoles | 1.537176 | 1.65E-08 | 5.58E-07 | 0.238947 | -2.06524 | down |
| 3-Methylglutaric acid | Fatty acyls[FA] | 1.616069 | 2.18E-05 | 0.000147 | 2.074841 | 1.053001 | up |
| Hydrocinnamic acid | Phenylpropanoic acids | 1.661432 | 6.34E-06 | 5.95E-05 | 3.009 | 1.589284 | up |
| Capryloylglycine | Amino acids, peptides, and analogues | 1.845187 | 7.94E-05 | 0.000394 | 5.846498 | 2.547573 | up |
| Pimelic acid | Organic acids | 1.734839 | 2.37E-07 | 4.19E-06 | 2.501037 | 1.322527 | up |
| Xanthurenic acid | Quinoline carboxylic acids | 1.714641 | 5.81E-05 | 0.000307 | 2.218835 | 1.149802 | up |
| Vitamin D2 | Vitamins | 1.919422 | 1.57E-08 | 5.57E-07 | 2.007296 | 1.005253 | up |
| L-Tryptophan | Amino acids | 1.210154 | 0.000131 | 0.000578 | 0.164845 | -2.60082 | down |
| trans-Cinnamic acid | Phenylpropanoids | 1.731642 | 1.18E-06 | 1.53E-05 | 2.977137 | 1.573926 | up |
| 3-Hydroxyanthranilic acid | Benzene and derivatives | 1.295972 | 4.00E-08 | 1.11E-06 | 2.3963 | 1.260808 | up |
| Benzoylformic acid | Benzene and derivatives | 1.377231 | 9.43E-07 | 1.31E-05 | 0.277741 | -1.84819 | down |
| 3-Coumaric acid | Polyketides[PK] | 1.007012 | 0.003595 | 0.009547 | 0.329044 | -1.60365 | down |
| Methylsuccinic acid | Fatty acyls[FA] | 1.974396 | 4.30E-08 | 1.12E-06 | 2.277211 | 1.187268 | up |
| 2,2-Dimethylsuccinic acid | Fatty acyls[FA] | 1.793609 | 5.55E-06 | 5.33E-05 | 2.145204 | 1.101115 | up |
| N6-Methyladenine | Purines and derivatives | 1.942819 | 1.08E-09 | 7.64E-08 | 2.231363 | 1.157926 | up |
| Phthalic acid | Benzene and derivatives | 1.769902 | 2.70E-07 | 4.57E-06 | 0.149418 | -2.74258 | down |
| 4-Nitrocatechol | Phenols and derivatives | 1.342764 | 0.006494 | 0.015658 | 3.699873 | 1.887476 | up |
| Palmitoleic acid | Fatty acyls[FA] | 2.263405 | 0.000154 | 0.000671 | 5.32079 | 2.41164 | up |
| Solanidine | Alkaloids | 1.622008 | 6.95E-05 | 0.000356 | 3.123343 | 1.643091 | up |
| 2-Amino-1,3,4-octadecanetriol | Sphingolipids[SP] | 1.99932 | 1.51E-15 | 5.89E-13 | 0.350803 | -1.51127 | down |
| Oleoyl-L-a-lysophosphatidic acid | Glycerophospholipids[GP] | 1.205781 | 0.015849 | 0.03228 | 0.106239 | -3.23462 | down |
| 2-Methylglutaric acid | Amino acids, peptides, and analogues | 1.821601 | 4.93E-05 | 0.000274 | 2.274448 | 1.185517 | up |
| 4-Aminobiphenyl | Benzene and derivatives | 1.035864 | 9.67E-06 | 8.01E-05 | 2.890681 | 1.53141 | up |
| Monobutyl phthalate | Benzene and derivatives | 1.827515 | 6.50E-06 | 5.95E-05 | 0.066389 | -3.91292 | down |
| Mono(2-ethylhexyl) phthalate (MEHP) | Benzene and derivatives | 1.320782 | 0.001399 | 0.004301 | 0.3298 | -1.60033 | down |
| a-Linolenoyl ethanolamide | Amines | 1.831843 | 3.01E-06 | 3.30E-05 | 4.699088 | 2.232381 | up |
| DL-Tryptophan | Indole and derivatives | 1.450301 | 3.75E-08 | 1.08E-06 | 0.2169 | -2.2049 | down |
| Neohesperidin | Flavonoids | 1.62191 | 2.28E-05 | 0.000152 | 0.059553 | -4.06969 | down |
| Avocadyne 1-acetate | Fatty acyls[FA] | 1.23763 | 0.000123 | 0.000549 | 0.468544 | -1.09374 | down |
| Benzophenone | Benzene and derivatives | 1.554267 | 0.015004 | 0.031046 | 3.615827 | 1.854326 | up |
| 2-Anisic acid | Benzene and derivatives | 2.1308 | 0.000115 | 0.000531 | 12.22607 | 3.611888 | up |
| 2,6-Pyridinecarboxylic acid | Pyridine and derivatives | 1.672421 | 0.008293 | 0.019144 | 8.828351 | 3.142144 | up |
| Harmane | Alkaloids | 1.259477 | 4.31E-07 | 6.58E-06 | 2.448042 | 1.291629 | up |
| Perillartine | Terpenoids | 1.429106 | 0.010168 | 0.022158 | 2.563395 | 1.358056 | up |
| Matairesinol | Lignans | 1.765683 | 4.24E-09 | 2.16E-07 | 0.148334 | -2.75308 | down |
| Corchorifatty acid F | Fatty acyls[FA] | 1.797537 | 0.000135 | 0.000593 | 2.458339 | 1.297684 | up |
| Eicosapentaenoic acid ethyl ester | Fatty acyls[FA] | 1.524404 | 4.64E-05 | 0.000262 | 2.533445 | 1.341101 | up |
| 3-Methoxysalicylic acid | Benzene and derivatives | 2.128922 | 3.86E-05 | 0.000229 | 12.03079 | 3.588659 | up |
| 5-Hydroxyindole | Indole and derivatives | 2.365806 | 4.11E-07 | 6.46E-06 | 50.16122 | 5.6485 | up |
| 11(Z),14(Z),17(Z)-Eicosatrienoic acid | Fatty acyls[FA] | 1.769467 | 2.13E-16 | 1.66E-13 | 2.081016 | 1.057288 | up |
| Pectolinarigenin | Flavonoids | 1.71305 | 0.000349 | 0.001345 | 54.18852 | 5.759915 | up |
| Dihydroresveratrol | Polyketides[PK] | 1.002747 | 0.000783 | 0.002721 | 0.292247 | -1.77474 | down |
| Loganic acid | Terpenoids | 1.173165 | 4.15E-06 | 4.14E-05 | 0.46517 | -1.10417 | down |
| Rhaponticin | Polyketides[PK] | 1.473604 | 4.21E-12 | 1.09E-09 | 0.38936 | -1.36082 | down |
| Retrorsine | Alkaloids | 1.105441 | 0.00358 | 0.00954 | 2.009901 | 1.007124 | up |
| Cannabidiolic acid | Polyketides[PK] | 1.078856 | 0.023475 | 0.044873 | 3.485631 | 1.80142 | up |
| Jervine | Alkaloids | 1.003896 | 8.36E-05 | 0.000412 | 0.20187 | -2.3085 | down |
| N-Arachidonoyl dopamine | 0 | 2.057472 | 9.73E-11 | 1.26E-08 | 0.108066 | -3.21001 | down |
| Adenosine | Nucleic acids | 1.508943 | 4.27E-10 | 3.69E-08 | 0.24906 | -2.00544 | down |
| 7-Methylguanine | Purines and derivatives | 1.333743 | 0.000876 | 0.003002 | 2.371091 | 1.245551 | up |
| N-Acetyl-DL-glutamic acid | Amino acids, peptides, and analogues | 1.657071 | 6.73E-10 | 5.24E-08 | 0.364281 | -1.45688 | down |
| Melatonin | Hormones and transmitters | 1.837636 | 1.44E-08 | 5.57E-07 | 0.150133 | -2.73569 | down |
| D-(+)-Glucosamine | Carbohydrates | 1.375204 | 0.005622 | 0.014099 | 0.100144 | -3.31985 | down |
| Menadione | Prenol lipids[PR] | 1.102053 | 1.10E-07 | 2.19E-06 | 0.299472 | -1.73951 | down |
| 3-Aminosalicylic acid | Benzene and derivatives | 1.068521 | 0.017074 | 0.034236 | 0.1671 | -2.58122 | down |
| Arachidonoyl serinol | Fatty acyls[FA] | 1.231307 | 9.19E-06 | 7.69E-05 | 0.291872 | -1.77659 | down |
| Polydatin | Polyketides[PK] | 1.370931 | 8.65E-11 | 1.26E-08 | 0.367976 | -1.44232 | down |
| Methyl cinnamate | Polyketides[PK] | 1.352115 | 3.03E-05 | 0.000193 | 2.423008 | 1.276799 | up |
| Demethoxyyangonin | Polyketides[PK] | 1.897769 | 0.014338 | 0.029825 | 3.71334 | 1.892717 | up |
| Glabrolide | Terpenoids | 1.012992 | 1.47E-05 | 0.000117 | 0.285941 | -1.80621 | down |
| Bilobalide | Terpenoids | 1.673546 | 5.72E-09 | 2.47E-07 | 0.110083 | -3.18333 | down |
| 2-Amino-3-methoxybenzoic acid | Benzene and derivatives | 1.265772 | 7.02E-06 | 6.21E-05 | 2.202102 | 1.138881 | up |
| Dihydrobiopterin; | Pteridines and derivatives | 1.857536 | 5.21E-09 | 2.38E-07 | 0.196002 | -2.35106 | down |
| N6-(L-1,3-Dicarboxypropyl)-L-lysine | Amino acids, peptides, and analogues | 1.758708 | 2.47E-07 | 4.27E-06 | 0.099567 | -3.32818 | down |
| sn-Glycero-3-phosphocholine | Glycerophospholipids[GP] | 1.596885 | 0.000359 | 0.001375 | 2.506668 | 1.325771 | up |
| 3-Methyladenine; | Purines and derivatives | 1.549568 | 4.48E-08 | 1.12E-06 | 0.374423 | -1.41726 | down |
| 2,3-Dimethylmaleate | Fatty acyls[FA] | 1.477354 | 1.35E-07 | 2.55E-06 | 2.102952 | 1.072416 | up |
| S-Formylglutathione; | Amino acids, peptides, and analogues | 1.175448 | 3.67E-05 | 0.000219 | 0.431031 | -1.21414 | down |
| 8-Amino-7-oxononanoate | Fatty acyls[FA] | 1.392184 | 0.002376 | 0.00665 | 2.404257 | 1.265591 | up |
| S-Acetyldihydrolipoamide | Fatty acyls[FA] | 1.001865 | 0.009979 | 0.021932 | 0.106117 | -3.23627 | down |
| Benzoin | Polyketides[PK] | 1.002762 | 0.000783 | 0.002721 | 0.292417 | -1.7739 | down |
| Luteolin | Flavonoids | 1.028997 | 0.000443 | 0.001651 | 0.296142 | -1.75564 | down |
| Xylobiose; | Carbohydrates | 1.220179 | 0.006026 | 0.01479 | 0.173543 | -2.52663 | down |
| Ethyl N-alpha-acetyl-tyrosinate; | Amino acids, peptides, and analogues | 1.538109 | 3.81E-07 | 6.17E-06 | 0.451028 | -1.14871 | down |
| Ornaline | Amino acids, peptides, and analogues | 1.07307 | 0.001823 | 0.005312 | 0.185234 | -2.43258 | down |
| Gibberellin A3 | Terpenoids | 1.660238 | 0.023781 | 0.045347 | 7.240404 | 2.85607 | up |
| Phylloquinone | Vitamins | 1.784245 | 1.24E-06 | 1.59E-05 | 2.701686 | 1.43386 | up |
| Sinapyl alcohol | Phenylpropanoids | 2.35754 | 0.000238 | 0.000974 | 20.06386 | 4.326527 | up |
| Protochlorophyllide | Metallotetrapyrroles | 1.441088 | 4.48E-06 | 4.41E-05 | 0.336308 | -1.57214 | down |
| 5-Oxo-1,2-campholide | Prenol lipids[PR] | 1.676804 | 5.64E-05 | 0.000305 | 2.62285 | 1.391136 | up |
| L-2-Hydroxyphytanate; | Prenol lipids[PR] | 1.578265 | 8.98E-05 | 0.000433 | 2.767429 | 1.468547 | up |
| L-Methionine sulfoximine; | Amino acids, peptides, and analogues | 2.384661 | 8.08E-06 | 6.91E-05 | 32.87422 | 5.038885 | up |
| Cholesteryl-beta-D-glucoside; | Sterol lipids[ST] | 1.731288 | 2.03E-05 | 0.00014 | 0.14294 | -2.80652 | down |
| 1-Oleoylglycerophosphocholine | Glycerophospholipids[GP] | 1.7588 | 7.78E-09 | 3.19E-07 | 0.149678 | -2.74006 | down |
| Isocorypalmine | Alkaloids | 1.664279 | 8.95E-06 | 7.57E-05 | 3.514708 | 1.813405 | up |
| 4alpha-Methyl-5alpha-cholest-7-en-3-one; | Sterol lipids[ST] | 1.276628 | 3.40E-05 | 0.000208 | 0.404635 | -1.30531 | down |
| 5-(4-Acetoxybut-1-ynyl)-2,2'-bithiophene; | Bi- and oligothiophenes | 1.354152 | 5.36E-07 | 7.86E-06 | 0.354603 | -1.49572 | down |
| (3S,4S)-3-Hydroxytetradecane-1,3,4-tricarboxylate; | Fatty acyls[FA] | 1.549698 | 0.000773 | 0.002709 | 6.708071 | 2.745898 | up |
| 3beta-Hydroxy-4beta-methyl-5alpha-cholest-7-ene-4alpha-carboxylate; | Sterol lipids[ST] | 1.69414 | 2.75E-09 | 1.53E-07 | 0.358403 | -1.48035 | down |
| (S)-Tetrahydroprotoberberine | Alkaloids | 2.052957 | 0.000104 | 0.000487 | 13.22483 | 3.725177 | up |
| Stizolobinate | Fungal toxins | 2.040863 | 6.76E-07 | 9.56E-06 | 5.799329 | 2.535886 | up |
| Elymoclavine; Dihydrolysergol; | Alkaloids | 2.077011 | 3.47E-07 | 5.74E-06 | 6.723934 | 2.749306 | up |
| Ajmaline; | Alkaloids | 2.018378 | 0.01835 | 0.036143 | 10.22036 | 3.353374 | up |
| p-Cymene; | Terpenoids | 1.480293 | 1.54E-05 | 0.00012 | 2.101867 | 1.071671 | up |
| Fusidic acid; | Sterol lipids[ST] | 1.508849 | 3.81E-06 | 3.91E-05 | 0.352821 | -1.50299 | down |
| 3-Hydroxyquinine; | Alkaloids | 1.81045 | 1.37E-09 | 8.89E-08 | 0.11775 | -3.0862 | down |
| Cassaine; | Alkaloids | 1.566659 | 0.001502 | 0.004565 | 3.873267 | 1.953551 | up |
| Karakoline | Alkaloids | 1.432979 | 0.0012 | 0.003794 | 2.818402 | 1.494878 | up |
| Methyllycaconitine; | Alkaloids | 1.008106 | 0.000367 | 0.001394 | 2.320244 | 1.214276 | up |
| Nudicauline; | Alkaloids | 1.664447 | 0.000371 | 0.0014 | 4.268156 | 2.093613 | up |
| Thalicsessine | Alkaloids | 1.257314 | 0.001481 | 0.004518 | 2.492383 | 1.317526 | up |
| Cimifugin; | Polyketides[PK] | 1.176198 | 0.004997 | 0.012663 | 2.456676 | 1.296708 | up |
| Borrerine; | Alkaloids | 1.483434 | 0.001085 | 0.003487 | 0.390205 | -1.3577 | down |
| Canthin-6-one | Alkaloids and derivatives | 1.690353 | 0.004196 | 0.010918 | 2.251867 | 1.171121 | up |
| Rhodojaponin IV | Terpenoids | 1.20092 | 0.014623 | 0.030337 | 4.361065 | 2.12468 | up |
| Sclareol | Terpenoids | 1.423225 | 0.000547 | 0.001981 | 2.99665 | 1.58335 | up |
| Evodiamine; | Alkaloids | 1.433683 | 5.14E-08 | 1.21E-06 | 0.289158 | -1.79007 | down |
| Gelsemicine; | Alkaloids | 1.255021 | 0.000986 | 0.003236 | 3.024597 | 1.596743 | up |
| (-)-Quebrachamine; Quebrachamine; | Alkaloids and derivatives | 1.773508 | 2.10E-10 | 2.33E-08 | 0.107783 | -3.21379 | down |
| Arctiopicrin; | Terpenoids | 1.253371 | 1.31E-05 | 0.000106 | 0.19531 | -2.35617 | down |
| Aromaticin; | Terpenoids | 1.161441 | 6.49E-06 | 5.95E-05 | 0.170949 | -2.54836 | down |
| (+-)-Carnegine | Alkaloids | 1.262232 | 0.026871 | 0.049901 | 2.579714 | 1.367211 | up |
| Cassythine | Alkaloids | 1.687727 | 1.01E-06 | 1.38E-05 | 0.142509 | -2.81087 | down |
| Erysonine; | Alkaloids | 1.752809 | 0.005772 | 0.014302 | 2.486632 | 1.314193 | up |
| Fetidine; | Alkaloids | 1.029977 | 5.64E-05 | 0.000305 | 0.477433 | -1.06663 | down |
| Hasubanonine; | Alkaloids | 1.356244 | 6.21E-05 | 0.000324 | 0.392846 | -1.34796 | down |
| Linifolin A; | Terpenoids | 1.607612 | 0.000126 | 0.00056 | 4.20097 | 2.070723 | up |
| Scorpioidin; | Terpenoids | 1.034345 | 2.00E-05 | 0.00014 | 0.289497 | -1.78838 | down |
| Laudanosine; | Alkaloids | 1.395003 | 9.68E-05 | 0.000456 | 0.157877 | -2.66313 | down |
| Scandoside methyl ester; | Terpenoids | 1.122429 | 0.001236 | 0.003877 | 0.26197 | -1.93252 | down |
| Cryptophorine; | Alkaloids | 1.205351 | 0.001924 | 0.005565 | 2.2813 | 1.189856 | up |
| Sinensetin | Flavonoids | 1.399538 | 6.66E-11 | 1.26E-08 | 0.471151 | -1.08574 | down |
| (-)-Glyceollin II; | Flavonoids | 1.396865 | 2.36E-08 | 7.07E-07 | 0.321908 | -1.63528 | down |
| Feruloylputrescine | Polyketides[PK] | 1.302241 | 0.000315 | 0.001232 | 2.502722 | 1.323498 | up |
| (+)-Elaeocarpine; | Alkaloids | 1.265914 | 1.37E-06 | 1.67E-05 | 0.286593 | -1.80292 | down |
| Pilosine; | Alkaloids | 2.072027 | 0.0047 | 0.011988 | 5.206922 | 2.380431 | up |
| Arborinine; | Alkaloids | 1.988872 | 7.21E-06 | 6.30E-05 | 6.633779 | 2.729831 | up |
| Atalaphylline; | Alkaloids | 1.508234 | 5.33E-06 | 5.18E-05 | 0.286504 | -1.80337 | down |
| Dictamnine; | Alkaloids | 1.409332 | 2.77E-06 | 3.11E-05 | 2.862163 | 1.517106 | up |
| Aspidinol; | Polyketides[PK] | 1.09802 | 0.000677 | 0.002417 | 2.221958 | 1.151832 | up |
| Robustine | Alkaloids | 1.01336 | 0.000122 | 0.000548 | 0.316132 | -1.6614 | down |
| Rutacridone epoxide; | Alkaloids | 1.128402 | 0.000652 | 0.002337 | 2.533381 | 1.341064 | up |
| Vasicinone; | Alkaloids | 1.111749 | 6.05E-05 | 0.000318 | 0.443122 | -1.17423 | down |
| Argyrolobine; | Alkaloids | 1.468964 | 7.41E-08 | 1.65E-06 | 0.153291 | -2.70566 | down |
| 5-(Heptadec-12-enyl)resorcinol; | Polyketides[PK] | 2.249366 | 4.15E-07 | 6.46E-06 | 9.822461 | 3.296085 | up |
| Tomatine; | Alkaloids | 1.676019 | 9.06E-05 | 0.000433 | 0.335069 | -1.57747 | down |
| Brugine; | Alkaloids | 1.041959 | 0.00248 | 0.006914 | 0.191089 | -2.38769 | down |
| Strobamine | Alkaloids | 1.716757 | 1.66E-05 | 0.000122 | 3.959315 | 1.985251 | up |
| Benalaxyl; | Amino acids, peptides, and analogues | 1.645778 | 1.73E-05 | 0.000124 | 3.547748 | 1.826903 | up |
| 16-Hydroxytabersonine; | Alkaloids | 1.032753 | 0.005984 | 0.014755 | 2.461205 | 1.299365 | up |
| 8-Epiiridodial glucoside; | Prenol lipids[PR] | 1.195777 | 0.001602 | 0.004814 | 3.06096 | 1.613984 | up |
| alpha-Ionone; | Terpenoids | 1.510546 | 3.87E-06 | 3.91E-05 | 0.365869 | -1.4506 | down |
| Acetylpseudotropine; | Alkaloids | 2.126228 | 1.37E-05 | 0.00011 | 8.630953 | 3.10952 | up |
| Tributyrin | Glycerolipids[GL] | 1.055571 | 1.97E-05 | 0.00014 | 0.316949 | -1.65768 | down |
| 4-Propylphenol; | Benzene and derivatives | 1.568174 | 9.32E-08 | 1.91E-06 | 0.205118 | -2.28547 | down |
| Dicyclohexylamine | Amines and derivatives | 1.121778 | 2.74E-06 | 3.11E-05 | 0.35451 | -1.4961 | down |
| 4,5-Dihydro-4-hydroxy-5-S-glutathionyl-benzo[a]pyrene; | Amino acids, peptides, and analogues | 1.67848 | 0.000386 | 0.001452 | 0.134817 | -2.89093 | down |
| 12-(2,3-Dihydroxycyclopentyl)-2-dodecanone; | Carbonyl compounds | 1.626466 | 2.09E-08 | 6.51E-07 | 0.241754 | -2.04839 | down |
| Apocholic acid; 3alpha,12alpha-Dihydroxy-5beta-chol-8(14)-en-24-oic acid; | Sterol lipids[ST] | 1.829087 | 3.67E-06 | 3.91E-05 | 2.809526 | 1.490327 | up |
| Chalcone; | Polyketides[PK] | 1.102667 | 0.000937 | 0.003156 | 2.585188 | 1.370269 | up |
| Chloroxanthin | Prenol lipids[PR] | 1.736824 | 1.73E-05 | 0.000124 | 3.037077 | 1.602683 | up |
| 12-Oxo-9(Z)-dodecenoic acid; | Fatty acyls[FA] | 1.587369 | 4.79E-08 | 1.17E-06 | 2.028918 | 1.020711 | up |
| (+)-7-Isomethyljasmonate; | Fatty acyls[FA] | 1.186313 | 2.79E-05 | 0.000179 | 0.483705 | -1.0478 | down |
| Volicitin | Fatty acyls[FA] | 1.451979 | 1.29E-06 | 1.59E-05 | 2.255902 | 1.173705 | up |
| 2-n-Propyl-4-oxopentanoic acid | Fatty acyls[FA] | 1.935986 | 1.83E-08 | 5.93E-07 | 2.305512 | 1.205087 | up |
| Kurarinol; | Polyketides[PK] | 1.739909 | 9.02E-05 | 0.000433 | 3.917016 | 1.969755 | up |
| Capsi-amide | Organic acids | 1.574088 | 0.000328 | 0.001275 | 2.696826 | 1.431263 | up |
| Cryptogenin | Sterol lipids[ST] | 1.185598 | 0.001063 | 0.003447 | 2.227932 | 1.155705 | up |
| 9beta-Pimara-7,15-diene; | Prenol lipids[PR] | 1.53226 | 1.96E-07 | 3.62E-06 | 0.422338 | -1.24353 | down |
| 4,4'-Diapophytofluene; | Prenol lipids[PR] | 1.016921 | 4.13E-05 | 0.000244 | 0.430282 | -1.21665 | down |
| Iriomoteolide 1a | Marine biotoxins | 1.302174 | 7.18E-08 | 1.64E-06 | 0.329244 | -1.60277 | down |
| Pseudoargiopinin III; | Venoms | 2.149161 | 0.000104 | 0.000487 | 6.119086 | 2.613316 | up |
| Capsiate; | Amino acid related compounds | 1.84297 | 3.35E-05 | 0.000207 | 3.602156 | 1.848861 | up |
| Nordihydrocapsaicin | Amino acid related compounds | 1.176227 | 0.000971 | 0.003201 | 2.30875 | 1.207112 | up |
| N-(4-Coumaroyl)-L-homoserine lactone; | Fatty acyls[FA] | 1.137371 | 0.003065 | 0.00828 | 2.162463 | 1.112675 | up |
| Phytyl phosphate; | Prenol lipids[PR] | 2.376905 | 0.001125 | 0.003587 | 29.48192 | 4.881759 | up |
| N-3-Hydroxyoctanoyl-L-homoserine lactone | Fatty acyls[FA] | 1.590856 | 2.04E-05 | 0.00014 | 0.399641 | -1.32322 | down |
| (3E)-4,8-Dimethylnona-1,3,7-triene; | Terpenoids | 1.084728 | 0.000968 | 0.003201 | 0.408703 | -1.29088 | down |
| Shibic acid | Fatty acyls[FA] | 1.323133 | 0.016858 | 0.033978 | 4.353924 | 2.122316 | up |
| (6Z,9Z)-Hexadecadienoic acid | Fatty acyls[FA] | 2.354262 | 7.49E-05 | 0.000373 | 5.656593 | 2.499933 | up |

Table S3. The 12 Min-optimal metabolic indices screening by MUVR

| **Index** | **Order** | **Rank** |
| --- | --- | --- |
| Acetylpseudotropine | 1 | 19.28 |
| 3-Methoxysalicylic acid | 2 | 21.44444 |
| Monobutyl phthalate | 3 | 33.90889 |
| Sinapyl alcohol | 4 | 65.89556 |
| Phytyl phosphate | 5 | 67.41333 |
| 3-Hydroxyanthranilic acid | 6 | 145.2378 |
| 1-Acetylaspidoalbidine | 7 | 238.5733 |
| Anacardic acid | 8 | 347.5333 |
| Eicosapentaenoic acid ethyl ester | 9 | 361.7489 |
| 8-Amino-7-oxononanoate | 10 | 392.9178 |
| 2-Amino-3-methoxybenzoic acid | 11 | 393.52 |
| 4-Dodecylbenzenesulfonic acid | 12 | 486.0156 |

Table S4. The 12 metabolic indices stability P-value of year and variety by ANOVA

| **Index** | **Years** | **Variety** |
| --- | --- | --- |
| Acetylpseudotropine | 0.549 | 0.375 |
| 3-Methoxysalicylic acid | 0.646 | 0.156 |
| Monobutyl phthalate | 0.792 | 0.621 |
| Sinapyl alcohol | 0.715 | 0.451 |
| Phytyl phosphate | 0.438 | 0.358 |
| 3-Hydroxyanthranilic acid | 0.529 | 0.170 |
| 1-Acetylaspidoalbidine | 0.603 | 0.156 |
| Anacardic acid | 0.892 | 0.048 |
| Eicosapentaenoic acid ethyl ester | 0.568 | 0.182 |
| 8-Amino-7-oxononanoate | 0.045 | 0.601 |
| 2-Amino-3-methoxybenzoic acid | 0.464 | 0.012 |
| 4-Dodecylbenzenesulfonic acid | 0.026 | 0.035 |
